# Daixie recipe ameliorates diet-induced MASH in mice *via* activating PI3K/AKT and Keap1/Nrf2 signaling

**DOI:** 10.3389/fendo.2026.1772033

**Published:** 2026-03-13

**Authors:** Xiaoli He, Jiawen You, Yanyan Deng, Yiren Hu, Shenglan Qi, Qian Li, Yunyi Yang, Xiaoxiao Qu, Yanting Shao, Xinyi Fu, Shiyu Yang, Zhiying Wang, Yunhao Li, Min Zheng, Wei Liu, Hongjie Yang, Guangbo Ge, Zheng Yao, Yanming He

**Affiliations:** 1 Department of Endocrinology, Center of Experimental Animals, Yueyang Hospital of Integrated Traditional Chinese and Western Medicine, Shanghai University of Traditional Chinese Medicine, Shanghai, China; 2 Shanghai Frontiers Science Center of Traditional Chinese Medicine (TCM) Chemical Biology, Institute of Interdisciplinary Integrative Medicine Research, Shanghai University of Traditional Chinese Medicine, Shanghai, China; 3 Department of Traditional Chinese Medicine, Zhuanqiao Community Health Service Center, Shanghai, China; 4 Key Laboratory of Liver and Kidney Diseases (Ministry of Education), Institute of Liver Diseases, Shuguang Hospital Affiliated to Shanghai University of Traditional Chinese Medicine, Shanghai, China

**Keywords:** Daixie recipe (DXR), hepatocyte apoptosis, Keap1/Nrf2 signaling pathway, metabolic dysfunction-associated steatohepatitis (MASH), oxidative stress, PI3K/Akt signaling pathway

## Abstract

**Background:**

Metabolic dysfunction-associated steatohepatitis (MASH) is a highly prevalent liver disease that can progress to cirrhosis and hepatocellular carcinoma. Despite its growing clinical burden, effective therapies remain limited. Daixie recipe (DXR), derived from *Danggui Shaoyao San* in *Jingui Yaolue*, has shown notable clinical efficacy in treating MASH, yet its underlying pharmacological mechanisms remain to be clarified.

**Purpose:**

To clarify the pharmacological effects of DXR and to elucidate the underlying mechanisms of DXR for treating MASH.

**Methods:**

UHPLC-Q-Orbitrap HRMS was used to identify the phytochemcials in DXR. The key targets and ingredients for combating MASH were explored by using a suite of *in vitro* and *in vivo* experiments, as well as molecular dynamics simulations and bioinformatics analysis. The MASH model was established in C57BL/6 male mice by feeding either a high-fat, high-fructose, high-cholesterol diet or a methionine- and choline-deficient (MCD) diet. ELISA and Western Blotting were used to measure liver tissue pathology, serum biochemistry, lipid synthesis enzymes, oxidative stress markers, pro-inflammatory mediators, apoptosis-related factors, and *p*-AKT1, Nrf2, and HO-1 expression. The effects of DXR on the PI3K/AKT pathway and Keap1/Nrf2 signaling were analyzed in free fatty acid-induced AML12 cells and HEK293-Nrf2-Luc cells.

**Results:**

DXR shows significant therapeutic efficacy in ameliorating hepatic steatosis and inflammation, as evidenced by the marked reduction in the levels of serum biomarkers (such as ALT, AST, TG, TC, and LDL-c). Phytochemcial analysis coupling with network pharmacology analyses identify key targets of DXR for treating MASH, including AKT1, EGFR, TP53, STAT3, and IL6, with biological processes related to oxidative stress. Further investigations show that DXR significantly up-regulates p-AKT1, Nrf2, and HO-1, suggesting that this recipe activates both the PI3K/AKT and Keap1/Nrf2 signaling pathways. It is also found that DXR down-regulates the expression levels of key lipogenic enzymes (such as FASN, SCD1, and ACC1) but upregulates CPT1a in hepatocytes. DXR also exhibits significant antioxidant and anti-inflammatory effects, as demonstrated by a marked reduction in MDA levels and inflammatory cytokines (TNF-α, IL-1β, and IL-6), along with the increased activity levels of both SOD and GSH-Px. Additionally, DXR reduces hepatocyte apoptosis by up-regulating Bcl-2 and down-regulating Bax. Molecular dynamics simulations and luciferase reporter assays show that four flavonoids in DXR are key active constituents to activate the PI3K/AKT and Keap1/Nrf2 signaling pathways. Specifically, quercetin promotes AKT1 phosphorylation, while four flavonoids activate Nrf2 signaling, with apigenin exhibiting the most potent effect.

**Conclusions:**

DXR effectively ameliorates MASH in a mouse model by reducing hepatic steatosis, inflammation, and oxidative stress. Its mechanism of action involves the suppression of lipid synthesis and the prevention of hepatocyte apoptosis, achieved by modulating key anti-inflammatory and antioxidant signaling pathways. Flavonoids (such as apigenin and quercetin) are identified as the key active ingredients in DXR responsible for activating Keap1/Nrf2 and PI3K/AKT signaling pathways.

## 1 Introduction

Metabolic dysfunction-associated steatohepatitis (MASH) is a chronic and progressive liver disease characterized by hepatic steatosis, hepatocyte injury (evidenced by ballooning), inflammation, and hepatic osteodystrophy (1). MASH often presents with subtle symptoms in its early stages, but as the disease progresses, patients may experience a loss of appetite, fatigue, discomfort in the liver region, and hepatomegaly. MASH is a severe form of metabolic dysfunction–associated steatotic liver disease (MASLD), and its global prevalence has been rising rapidly in recent years (2). MASLD is marked by excessive hepatic fat accumulation, which may progress from simple steatosis (without significant inflammation or fibrosis) to MASH and eventually lead to hepatic fibrosis, cirrhosis, or even hepatocellular carcinoma (3). MASH has become an increasingly significant contributor to end-stage liver disease, posing substantial health risks and economic burdens on patients, families, and society (4). Although various studies aim to understand MASH pathogenesis, its key mechanisms remain elusive. The popular “multiple-hit” hypothesis suggests that excessive hepatic fat accumulation, insulin resistance, oxidative stress, lipid peroxidation, increased pro-inflammatory mediators, and other pathological factors contribute to the onset and progression of MASH (5, 6). Given the complex pathophysiology of MASH, pharmacologic therapies have recently progressed, including the approval of resmetirom, yet effective treatment options remain limited and are not universally effective.

Daixie recipe (DXR) is a modification of the classical Chinese medicine formula *Danggui Shaoyao San* (DSS) in Jingui Yaolue, is composed of eight medicinal herbs: *Angelica sinensis* (Oliv.) Diels, *Poria cocos* (Schw.) Wolf, *Ligusticum chuanxiong* Hort., leaves of *Morus alba* L., *Astragalus membranaceus* (Fisch.) Bunge, *Prunus mume* Siebold & Zucc., *Ligustrum lucidum* Ait., and *Ganoderma lucidum* (Leyss. ex Fr.) Karst. Recent studies have suggested that DSS may intervene in metabolic disorders and liver fibrosis. DSS alleviated CCl_4_-induced hepatic fibrosis through modulation of gut microbiota, short-chain fatty acids, and bile acid metabolism, thereby protecting the intestinal barrier and improving liver pathology (7). Similarly, in a zebrafish model that DSS significantly ameliorated lipid metabolism disorders *via* activation of the PPAR signaling pathway, resulting in reduced hepatic lipid accumulation and improved hyperlipidemia-associated symptoms (8). Moreover, DSS improved gut microbiota dysbiosis, attenuated inflammation, and restored hepatic lipid homeostasis in fructose-fed rats, suggesting a mechanistic role involving regulation of intestinal flora, hepatic gene expression, and metabolic pathways (9). Additionally, *Morus alba* leaves and *Astragalus membranaceus* have been shown to alleviate hepatic lipogenesis and fibrosis, reduce oxidative stress, and downregulate inflammatory mediators in mice fed a high-fat diet (10, 11), and *Prunus mume*, rich in polyphenols and flavonoids, effectively protects cells from oxidative damage (12). However, the pharmacological effects and mechanisms of DXR in MASH remain unclear.

To further clarify the therapeutic efficacy and mechanisms of DXR against MASH, a methionine- and choline-deficient (MCD) diet model was used for preliminary dose–response evaluation, while a metabolically relevant diet-induced MASH mouse model (HFHCD) was employed as the primary *in vivo* model for mechanistic investigation and conclusion drawing. In addition, a free fatty acid-stimulated AML12 cell model were used to evaluate its anti-MASH effects. Meanwhile, UHPLC-Q-Orbitrap HRMS-based phytochemical profiling, bioinformatics analyses, molecular docking and molecular dynamics simulations, as well as a stable Nrf2-luciferase reporter cell assay were employed to systematically uncover the bioactive components in DXR and related key signaling or targets. By integrating *in vivo*, *in vitro*, and computational approaches, this study highlights the regulatory effects of DXR on the PI3K/AKT and Keap1/Nrf2 signaling pathways, providing new insights into potential therapeutic strategies for MASH.

## 2 Materials and methods

### 2.1 Preparation of the DXR

The herbal decoction pieces of *Poria cocos* (Schw.) Wolf. (PC), the rhizomes of *Angelica sinensis* (Oliv.) Diels (AS), the rhizomes of *Ligusticum chuanxiong* Hort. (LC), the leaves of *Morus alba* L. (MA), the rhizomes of *Astragalus membranaceus* (Fisch.) Bunge (AM), the fruits of *Prunus mume* Siebold & Zucc. (PM), the fruits of *Ligustrum lucidum* Ait. (LL), and *Ganoderma lucidum* (Leyss. Ex Fr.) Karst. (GL) (Cat. No. 210819) were purchased from Hongqiao Chinese Herbal Decoction Pieces Co., Ltd. (Shanghai, China). The standard doses for DXR are as follows: PC 15 g, AS 9 g, LC 6 g, MA 9 g, AM 15 g, PM 9 g, LL 9 g, and GL 9 g per day (for a 60 kg adult, based on the weight of crude drugs) (**Supplementary Table 1**). The plant materials were authenticated by Prof. Wei Liu. Voucher specimens (Nos. 181106-1, 190215-1, 181227-1, 190713-4, 181022-2, 190901-2, 181223-2, and 190328-1) were deposited at the Department of Chinese Medicine Preparations, Shuguang Hospital Affiliated to Shanghai University of Traditional Chinese Medicine.

A total of 500 g of DXR pieces were soaked in 10 volumes of distilled water at room temperature for 30 min, heated to boiling, and decocted for 30 min. The decoction was filtered, and the filtrate was collected. The residue was decocted again in 8 volumes of distilled water for 30 min and filtered. Both filtrates were combined, filtered through gauze while hot, and concentrated under reduced pressure to a thick paste, which was stored at −80°C until further use.

### 2.2 Phytochemical profiling of DXR by UHPLC-Q-Orbitrap HRMS

The phytochemicals in DXR were identified using UHPLC-Q-Orbitrap HRMS (Thermo Fisher Scientific Inc., NY, USA). The UHPLC system employed was Thermo Scientific Dionex Ultimate 3000, controlled by Chromeleon 7.2 Software. The cooling autosampler was maintained at 10°C, protected from light, and the column heater was set at 45°C. A Waters ACQUITY UPLC BEH C18 column (2.1 × 100 mm, 1.7 μm) was utilized. The mobile phase consisted of A (methanol) and B (0.1% formic acid) at a flow rate of 0.3 ml/min and followed a gradient elution: 0–2.0 min (4% A), 2.0–6.0 min (4–12% A), 6.0–38.0 min (12–70% A), 38.0–38.5 min (70% A), 38.5–39.0 min (70–95% A), 39.0–43.0 min (95% A), 43.0–45.0 min (4% A). The injection volume was 2 μL. The Q-Orbitrap mass spectrometer was connected to the UHPLC system through heated electrospray ionization and controlled by Xcalibur 4.1 software for data capture and analysis. The electrospray ionization source was operated and optimized in negative ionization mode. The optimized mass spectrometry parameters were: capillary temperature: 320°C; sheath gas (N_2_) flow rate: 35 arbitrary units; auxiliary gas (N_2_) flow rate: 10 arbitrary units; sweep gas flow rate: 0 arbitrary units; spray voltage: 2.8 kV; S-lens RF level: 50 V; auxiliary gas heater temperature: 300°C; scan mode: full MS: scan range: 80–1200 m/z.

### 2.3 Experimental animals, diets, and treatment

Twenty-four male C57BL/6 mice (6 weeks old, weighing 18–22g) were obtained from Jihui Experimental Animal Breeding Co., Ltd, Shanghai, CHN. The animals were acclimated for one week at the Animal Experimental Center of Yueyang Hospital, affiliated with Shanghai University of Traditional Chinese Medicine (License No. SYXK 2018-0040). After acclimation, mice were randomly assigned using a simple randomization method into four groups: the methionine-choline sufficient (MCS) group, the methionine-choline deficient (MCD) group, the MCD + DXR low-dose group (DXR-L, 0.3 g/kg), and the MCD + DXR high-dose group (DXR-H, 0.6 g/kg). The MCS group was fed with a methionine-choline sufficient diet (Research Diets, Cat. No. A02082003B), while the other three groups were fed with a methionine-choline deficient diet (Research Diets, Cat. No. A02082002BR) for 6 weeks (13). From the third week onward, mice in the DXR-L and DXR-H groups received daily oral gavage of DXR at 0.3 g/kg and 0.6 g/kg, respectively, for a duration of 3 weeks, whereas the MCS and MCD groups were given an equivalent volume of vehicle.

In another model, twenty-four male C57BL/6 mice (6 weeks old, weighing 18–22g) were obtained from Jihui Experimental Animal Breeding Co., Ltd, Shanghai, CHN. Subsequently, the mice were randomly divided into two groups: the chow diet (Normal) group (*n* = 6) and the high-fat high-fructose high-cholesterol diet (HFHCD) group (*n* = 18). These groups were fed a chow diet or HFHCD for a duration of 24 weeks. The HFHCD group received high fructose corn syrup (23.1 g/L fructose + 18.9 g/L glucose) supplemented with water, while the Normal group was provided with regular water. The HFHCD, consisting of 40% kcal fat, 40% kcal carbohydrate, 20% kcal protein, and 2% cholesterol, was utilized to induce the MASH mouse model as previously described (14). The HFHCD (Cat. No. XT310) was procured from Xietong Pharmaceutical Bioengineering Co., Ltd, Nanjing, CHN. Chow diets (Cat. No. P1101F) were obtained from Pulutong Biotechnology Co., Ltd, Shanghai, CHN.

After 20 weeks of HFHCD feeding, mice in the HFHCD group were randomly reallocated into three subgroups (HFHCD, HFHCD + DXR, and HFHCD + semaglutide (SMG); n = 6 per group), and subsequently treated with 0.6 g/kg DXR extract per day *via* gavage administration or 13.3 μg/kg SMG per day *via* subcutaneous injection for 4 weeks. SMG was employed as a positive control drug, as previously reported (15).

### 2.4 Hematoxylin-eosin and oil red O staining

H&E staining: The paraffin sections of the liver tissue were initially dewaxed and rehydrated. Subsequently, the sections were stained with hematoxylin dye for 15 minutes and eosin dye for 1 minute. The stained sections were then scanned using a Leica SCN400 Slide Scanning System (Leica Inc., Wetzlar, GER). Histological assessments, including NAS scoring and quantitative histological analyses, were performed by investigators blinded to group allocation.

ORO staining: The frozen sections of the liver tissue were stained with Oil Red O dye for 20 minutes. After three washes with a PBS solution for 5 minutes each, the sections were subsequently stained with hematoxylin dye for 5 minutes. Following this, the sections were sealed with glycerol gelatin and scanned using a Leica SCN400 Slide Scanning System.

### 2.5 Collection of pathological genes in MASH patients

Pathological genes associated with MASH were acquired and compiled from the GeneCards database (https://www.genecards.org/) and the Online Mendelian Inheritance in Man database (OMIM, https://www.omim.org/). Retrieval was conducted using the keywords “Metabolic dysfunction-associated steatohepatitis”, “MASH”, “non-alcoholic steatohepatitis”, and “NASH”.

### 2.6 Screening of the components and targets of DXR in the treatment of MASH

The active components of DXR were identified using UHPLC-Q-TOF-MS analysis. Potential therapeutic targets of these components were determined through the SwissTargetPrediction tool (http://www.swisstargetprediction.ch/). The SMILES strings of each component were entered into SwissTargetPrediction, with targets identified at “Probability > 0” considered potential therapeutic targets. Target data for these compounds were also directly obtained from the TCMSP database (with OB ≥ 30% and DL ≥ 0.18), and results from both sources were combined. Structure files for each active component were downloaded from DrugBank (https://go.drugbank.com/) and PubChem (https://pubchem.ncbi.nlm.nih.gov/), while potential target names were gathered from the UniProt (https://www.uniprot.org/) and GeneCards databases. Finally, a Venn diagram was generated using an online tool (http://bioinformatics.psb.ugent.be/webtools/Venn/).

### 2.7 Bioinformatic analysis and screening of high-potential targets

In this study, the primary treatment target hub, M&D, was identified by overlapping DXR treatment targets with pathological genes linked to MASH. The intersected hub was visualized using an online Venn diagram tool (http://bioinformatics.psb.ugent.be/webtools/Venn/). The network interaction of M&D was then mapped using STRING, an online database for protein-protein interaction networks and functional enrichment analysis (http://string-db.org).

To examine core gene functions, a Gene Ontology (GO) enrichment analysis of M&D was conducted, covering molecular function, biological process, and cellular component categories. Kyoto Encyclopedia of Genes and Genomes (KEGG) enrichment analysis was also performed to explore the functions of key signaling pathways. Both GO and KEGG analyses were visualized with R software. Finally, topological analysis of M&D, focusing on core genes, was carried out using Cytoscape software.

### 2.8 Determination of the MDA, GSH-Px, and SOD levels in liver tissue

Liver tissues were homogenized in ice-cold PBS (1:9, w/v), and the supernatants were collected after centrifugation. Malondialdehyde (MDA, Cat. No. SBJ-M0411), glutathione peroxidase (GSH-Px, Cat. No. SBJ-M0019), and superoxide dismutase (SOD, Cat. No. SBJ-M0412) levels were determined using commercial ELISA kits (SenbeiJia Biological Technology Co., Ltd., Nanjing, CHN) according to the manufacturer’s instructions.

### 2.9 Terminal deoxynucleotidyltransferase-mediated dUTP-biotin nick end labeling staining

A TUNEL staining kit (Cat. No. C1098, Beyotime) was used to prepare paraffin slides, which were dewaxed, rehydrated, and treated with 20 μg/mL proteinase K solution for 30 minutes at room temperature. After washing three times with PBS, 50 μl of TUNEL working solution was applied to each sample and incubated at 37°C in the dark for 1 hour. Following the addition of the stopping solution and three PBS washes, DAB solution was used to visualize positively stained cells. The slides were counterstained with hematoxylin for 20 seconds and mounted with neutral resin. High-resolution images were captured with a Leica SCN400 Slide Scanning System, and TUNEL-positive cells were quantified using ImageJ (NIH, USA).

### 2.10 Immunofluorescence & immunohistochemical staining

Immunofluorescence staining: The frozen sections were fixed at 4°C with acetone for 20 minutes. Subsequently, the sections were permeated with 0.5% Triton-X 100 for 15 minutes, blocked with 10% goat serum in PBS for 30 minutes at room temperature, and incubated with phosphorylated AKT1 primary antibodies (**Supplementary Table 2**) at 4°C for 12 hours. FITC-conjugated secondary antibodies (**Supplementary Table 2**) were employed to visualize the primary antibodies, and DAPI was used to visualize the nuclei. The sections were sealed with an anti-fluorescence quenching solution and observed using an FV10C-W3 Laser Confocal Microscope (Olympus Inc., TKY, JPN).

Immunohistochemistry: Paraffin sections were dewaxed, rehydrated, and subjected to antigen retrieval in a citric acid solution using microwave heating. The slides were then treated with 3% H_2_O_2_-methanol solution for 30 minutes and blocked with 10% goat serum at room temperature for 30 minutes. Afterward, samples were incubated overnight at 4°C with either Bax (Abcam, ab32503, 1:300) or Bcl-2 (Abcam, ab182858, 1:200) antibodies. Following three PBS washes, an HRP-secondary antibody (Gene Tech, Cat. No. GK500705) was applied for 30 minutes at room temperature. The DAB solution was used to stain positive areas, and nuclei were counterstained with hematoxylin. High-resolution images were obtained with a Leica SCN400 Slide Scanning System, and Bax and Bcl-2 positive areas were semiquantitatively analyzed using Aperio Scope software (Leica, Wetzlar, GER).

### 2.11 Molecular docking simulations

The top four active components of DXR were identified using UHPLC-Q-Orbitrap HRMS and further screened through bioinformatic analysis, and their mol2 files were downloaded from the PubChem database (https://pubchem.ncbi.nlm.nih.gov/). The PDB files of the M&D core targets were obtained from the RCSB PDB database (https://www.rcsb.org/). Initially, the protein structure was prepared by removing water and small molecules, adding hydrogen atoms, repairing amino acid residues, and minimizing energy using PyMOL software. Subsequently, the mol2 files were imported into AutoDock Vina software and saved as pdbqt format files. Finally, the active components were semi-flexibly docked into the catalytic active domain of the target protein using AutoDock Vina. The binding energy was calculated, and the 3D diagram of the ligand-receptor docking was generated.

### 2.12 Molecular dynamics simulations

The top four active components of the metabolite DXR in mol2 format were downloaded from PubChem, while the protein crystal structures of PI3K and Keap1 (in PDB format) were retrieved from the RCSB PDB database. Ligands were processed using CGenFF to generate topology files, and molecular dynamics simulations were performed using Gromacs 2022.6. A cubic box was created, filled with the SPC water model, and 0.15M KCl or NaCl solution was added to neutralize the charge. The system was equilibrated under NPT and NVT ensembles using the CHARMM36 force field, with a temperature of 300K and a simulation time of 100ns. Visualization was carried out using R software.

### 2.13 Reagents and drugs

Triglyceride (TG) (Cat. No. A110-1-1), total cholesterol (TC) (Cat. No. F002-1-1), alanine aminotransferase (ALT) (Cat. No. C009-2-1), aspartate aminotransferase (AST) (Cat. No. C010-2-1), and low-density lipoprotein cholesterol (LDL-c) test kits (Cat. No. C009-2-1), along with hematoxylin-eosin (Cat. No. D006-1-1), and oil red O staining kits (Cat. No. D027-1-1), were supplied by Jiancheng Bioengineering Institute (Nanjing, China). Fructose (Cat. No. 1040071000), glucose (Cat. No. G8270), oleic acid (Cat. No. O1008), and palmitic acid (Cat. No. P0500) were procured from Sigma-Aldrich (Shanghai, China). The Total RNA Extractor (TRIzol) kit (Cat. No. B511311) was obtained from Sango Biotechnology (Shanghai, China), and the ReverTra Ace^®^ qPCR RT Master Mix with gDNA Remover kit (Cat. No. FSQ-301) from Toyobo (Shanghai, China). Beyotime Biotechnology Inc. (Shanghai, China) provided the RIPA lysis buffer (Cat. No. P0013B), protease and phosphatase inhibitors (Cat. No. P1045), and dexamethasone (Cat. No. ST1254-50mg). DMEM/high glucose medium (Cat. No. 10569010), fetal bovine serum (Cat. No. 16140071), insulin-transferrin-selenium G (ITS-G) (Cat. No. 41400045), and penicillin-streptomycin solution (Cat. No. 15140122) were obtained from Gibco (Thermo Fisher Scientific, Shanghai, China). The Steady-Glo^®^ Luciferase Assay kit (Cat. No. E2510) was purchased from Promega Corporation (WI, USA).

Semaglutide (Cat. No. 202110AJK1, MPN. SJ20210014) was purchased from Novo Nordisk Pharmaceuticals Co., Ltd. (Tianjin, CHN). Sulforaphane (Cat. No. 574215) was obtained from Sigma-Aldrich (Merck Ltd., Shanghai, CHN). Quercetin (Cat. No. BP1187), kaempferol (Cat. No. BP0820), apigenin (Cat. No. BP0177) and luteolin (Cat. No. BP0896) were purchased from Purfield Biotechnology Co., Ltd. (Chengdu, CHN).

### 2.14 Construction of stable transfection cells, cell culture, and Nrf2 reporter assay

The HEK293 (CVCL_0045; Cat. No. SCSP-5500) and AML12 (CVCL_0140; Cat. No. SCSP-550) cell lines were obtained from the Chinese Academy of Sciences Cell Bank (Shanghai, China). The HEK293-Nrf2-luc cell line was established through lentiviral stable transfection of the pGMLV-Nrf2-luc plasmid. The pGMLV-Nrf2-luc plasmid, designed by Genomeditech Biotechnology Co., Ltd. (Shanghai, CHN), contains the Nrf2 promoter-luciferase reporter gene construct driven by multiple antioxidant response elements (AREs). Subsequently, a stable transfected HEK293-Nrf2-luc cell line was generated *via* antibiotic screening.

The stable transfected HEK293-Nrf2-luc cell line was cultured in DMEM medium supplemented with 10% fetal bovine serum and 1% penicillin-streptomycin solution. AML12 cells were cultured in DMEM medium containing 10% fetal bovine serum, 1% ITS-G, 0.1% dexamethasone, and 1% penicillin-streptomycin solution. AML12 cells were incubated with 300 μM free fatty acid (oleic acid: palmitic acid = 2: 1 [*v*/*v*]) for 24 h to establish the *in vitro* model of hepatosteatosis. The culture conditions were maintained at a constant temperature of 37 °C in a humidified atmosphere of 95% air and 5% CO_2_.

For experimentation, HEK293-Nrf2-luc cell lines were initially seeded in 96-well plates at a density of 8 × 10^3^ cells per well and allowed to grow overnight. Quercetin, kaempferol, apigenin, and luteolin were added at concentrations of 1, 2, 5, and 15 μM, with sulforaphane (SFN, a well-established pharmacological activator of Nrf2) included as a positive control (16). The negative control group was treated with the vehicle DMSO alone, with the final concentration of DMSO in all experiments kept below 1% (*v*/*v*). Following a 24-hour incubation, luciferase activity was measured using the Steady-Glo^®^ Luciferase Assay following the manufacturer’s instructions. Luciferase activity was measured on a Spectramax M3 (Molecular devices, USA) with luciferase production as the readout. Data were presented as the percentage of fold induction of the test compound against the vehicle control group.

### 2.15 Total mRNA isolation, reverse transcription, and qPCR

Following the treatment, liver tissues were homogenized and treated with TRIzol. The total mRNA was then isolated using TRIzol methods in accordance with the manufacturer’s instructions. Reverse transcription and quantitative real-time PCR were conducted as previously described (17). The primers for *Tnf-α*, *Il-1β*, *Il-6*, *Fasn*, *Srebp1-c*, *Scd1*, *Acc1*, and *Cpt1*a for mice were synthesized by Sango Biotechnology Co., Ltd. (Shanghai, CHN) (**Supplementary Table 3**).

### 2.16 Protein isolation and western blotting

After treatment, liver tissues or AML12 cells were lysed with RIPA buffer containing 2% protease and phosphatase inhibitors and homogenized. Protein concentration was measured using a BCA protein assay kit (Cat. No. A55865) procured from Thermo Fisher Scientific (Grand Island, NY, USA). The homogenate was then mixed with SDS-PAGE loading buffer and heated at 100°C for 10 minutes. The target protein was isolated *via* gel electrophoresis, followed by transmembrane procedures and incubation with primary and secondary antibodies.

The phosphorylated AKT1 primary antibody (Cat. No. 44-621G) was sourced from Thermo Fisher Scientific (Grand Island, NY, USA), while the AKT1 (Cat. No. sc-5298) and HO-1 (Cat. No. sc-136960) primary antibodies were from Santa Cruz Biotechnology (Shanghai, CHN). The Nrf2 primary antibody (Cat. No. ab31163) was obtained from Abcam Shanghai (Shanghai, CHN). HRP-conjugated and FITC-conjugated secondary antibodies were purchased from Beyotime Biotechnology (Shanghai, CHN). The detailed list of antibodies is shown in **Supplementary Table 2**.

### 2.17 Statistical analysis

The data were presented as mean ± standard deviation. Statistical analyses were performed using one-way ANOVA followed by Tukey’s *post hoc* multiple-comparison test. A significance level of *P* < 0.05 was considered statistically significant.

## 3 Results

### 3.1 DXR ameliorates hepatosteatosis and inflammation in diet-induced MASH mice

**Figure 1:**
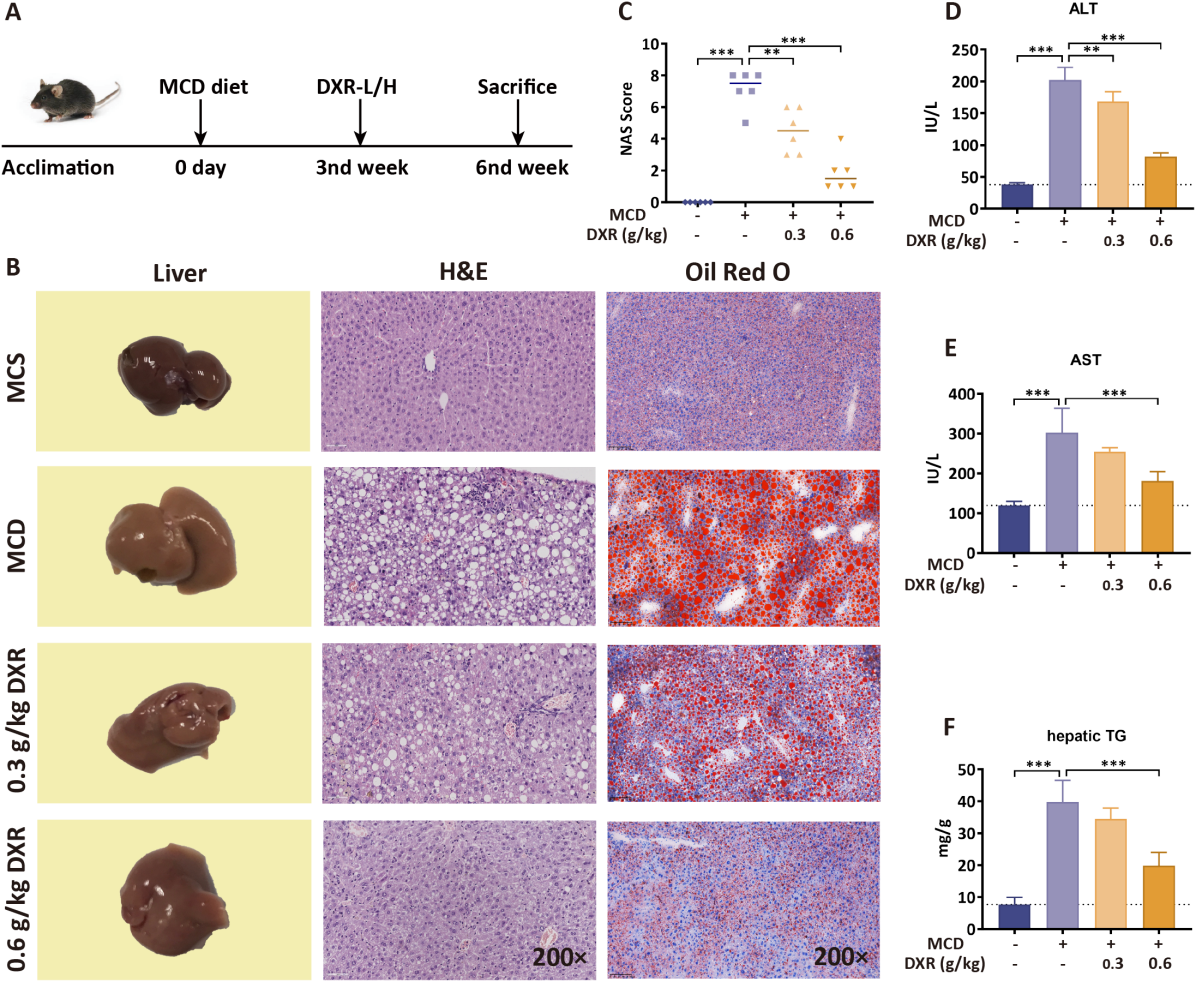
DXR alleviates hepatic steatosis and injury in MCD diet-induced MASH mice. **(A)** Representative liver morphology, H&E staining, and Oil Red O staining (200×) showing reduced steatosis after DXR treatment. **(B)** NAS score was decreased by DXR in a dose-dependent manner. **(C)** Hepatic NAS tc tal score was significantly reduced by DXR. **(D, E)** Serum ALT and AST levels were significantly reduced by DXR. **(F)** Hepatic TG content was lowered after DXR administration. ^*^P < 0.05; ^**^P < 0.01; ^***^P < 0.001.

First, an MASH model was established by feeding C57BL/6J mice a MCD diet for 6 weeks. Beginning at week 3, mice received DXR at varying doses for 3 consecutive weeks, to determine an effective *in vivo* dosing regimen for DXR (**Figure 1A**). H&E and Oil Red O staining revealed that DXR markedly alleviated hepatic steatosis, lobular inflammation, and fibrosis in a dose-dependent manner, with the high dose (0.6 g/kg) showing the most pronounced improvements in histopathology, NAS score, serum ALT levels, and hepatic TG content (**Figures 1B–E**).

**Figure 2:**
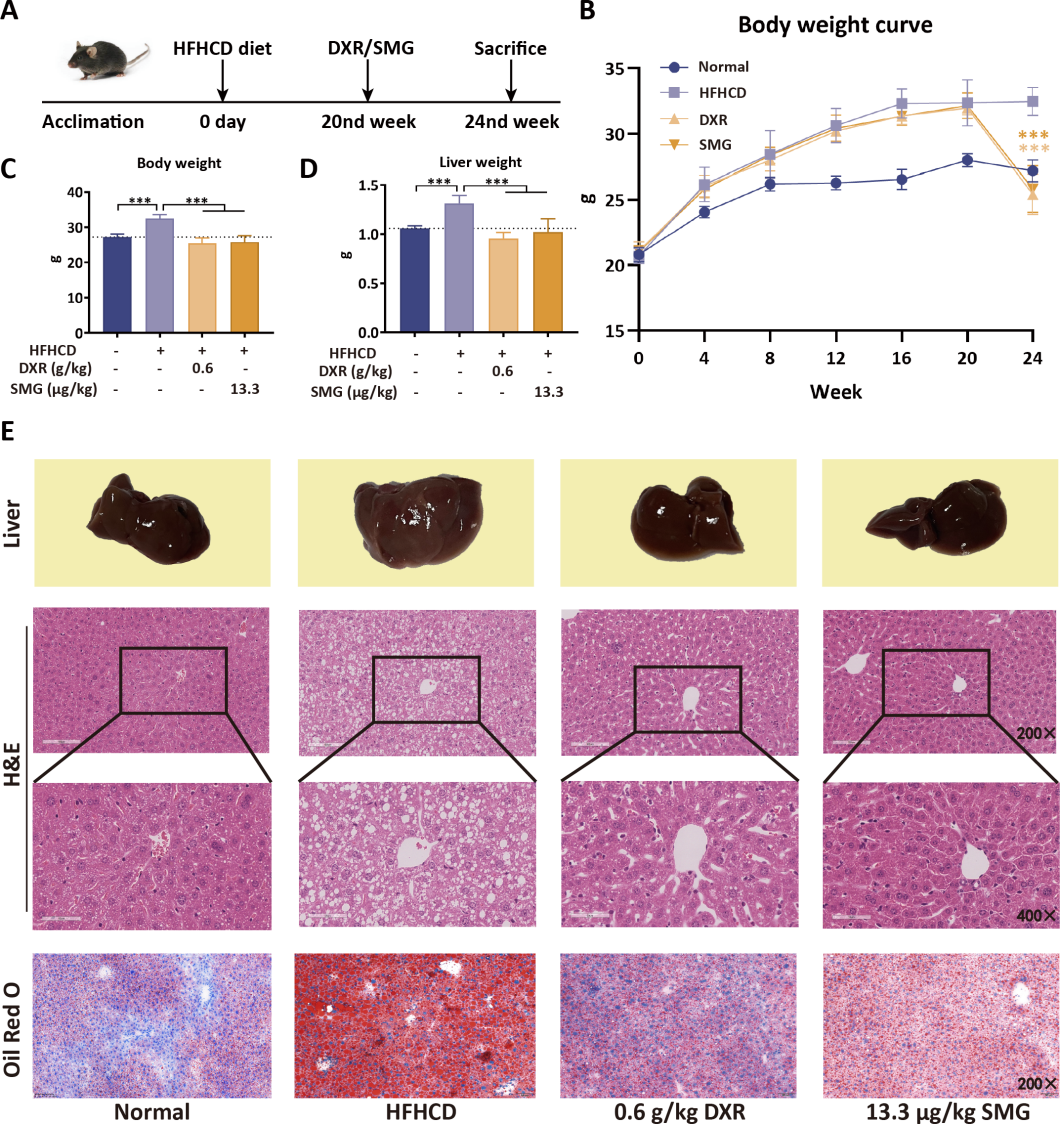
DXR improves liver pathology in HFHCD-induced MASH mice. **(A)** Experimental scheme of HFHCD-induced MASH and DXR treatment. **(B–D)** DXR reduced body weight gain and liver weight. **(E)** Liver appearance and H&E (200×, 400×)/Oil Red O (200×) staining showed decreased steatosis and inflammation. ^***^P < 0.001 vs. HFHCD.

**Figure 3:**
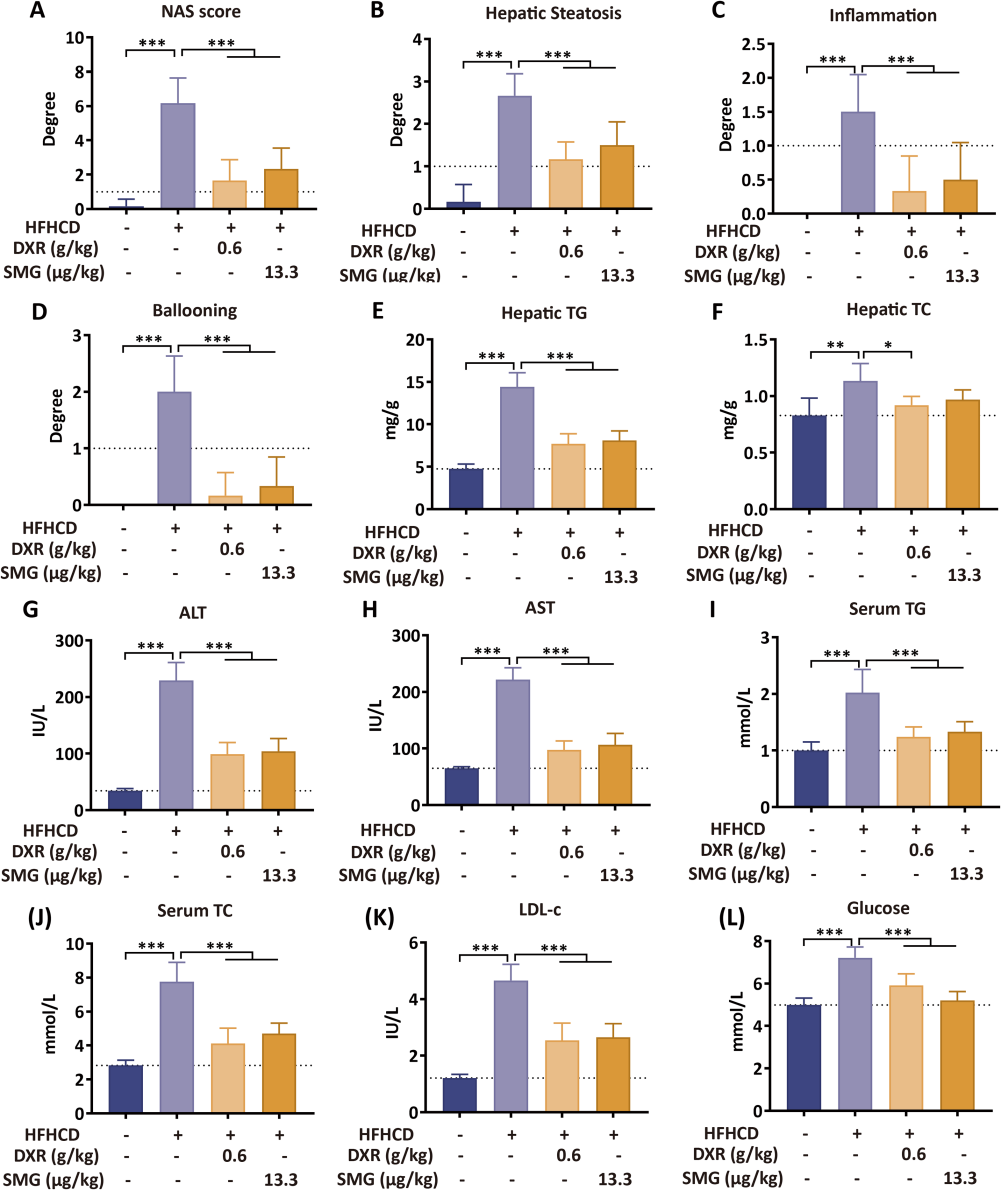
DXR reduces hepatic lipid accumulation and serum biochemical markers. **(A–D)** NAS, steatosis, inflammation, and ballooning scores were decreased by DXR. **(E–F)** Hepatic TG and TC levels were reduced. **(G–L)** Serum ALT, AST, TG, TC, LDL-c, and glucose were decreased after DXR treatment. ^*^P < 0.05; ^**^P < 0.01; ^***^P < 0.001 vs. HFHCD.

Based on this optimal dosing, a second, diet-induced MASH model was established by feeding C57BL/6 mice a HFHCD diet with fructose-enriched drinking water for 24 weeks. Treatment with DXR for the final 4 weeks significantly ameliorated hepatic steatosis, inflammation, and fibrosis in this chronic metabolic model (**Figure 2A**). The body and liver weights of the mice in the HFHCD group initially increased, followed by a decrease after treatment with DXR and SMG (**Figures 2B–D**). The livers of mice in the HFHCD group exhibited a pale and swollen appearance, which returned to a pinkish hue after treatment with DXR or SMG (**Figure 2E** **Liver**). Histopathological examination of the liver revealed that DXR and SMG markedly improved hepatosteatosis, lobular inflammation, and ballooning degeneration (**Figures 2E**, **3A–D**). Regarding lipid accumulation, DXR and SMG significantly reduced the hepatic levels of TG and TC compared to the HFHCD group (**Figures 3E, F**). Serum biochemical analysis further revealed that DXR and SMG reduced the serum levels of ALT, AST, TG, TC, LDL-c, and glucose compared to the HFHCD group (**Figures 3G–L**). These results from both the MCD-induced and HFHCD-induced models indicate that DXR exhibits consistent therapeutic potential against MASH of different etiologies.

### 3.2 Phytochemical profiling of DXR by UHPLC-Q-orbitrap HRMS

The UHPLC-Q-Orbitrap HRMS detection of the DXR extract revealed a total of 175 active compounds, including 73 in the ESI+ mode and 102 in the ESI- mode (**Supplementary Figure 1**, **Supplementary Table 4**). The components primarily derived from *Astragalus membranaceus* (Fisch.) Bunge (AM) number 62, those from *Ligusticum chuanxiong* Hort. (LC) total 33, from *Ligustrum lucidum* Ait. (LL) are 35, from *Ganoderma lucidum* (Leyss. Ex Fr.) Karst. (GL) amount to 30, from *Morus alba* L. (MA) are 28, from *Prunus mume* Siebold & Zucc. (PM) total 12, from *Angelica sinensis* (Oliv.) Diels (AS) number 4, and from *Poria cocos* (Schw.) Wolf (PC) are 4 (**Supplementary Figure 2**).

### 3.3 Bioinformatic analysis of the core gene hub and active components of DXR against MASH

**Figure 4:**
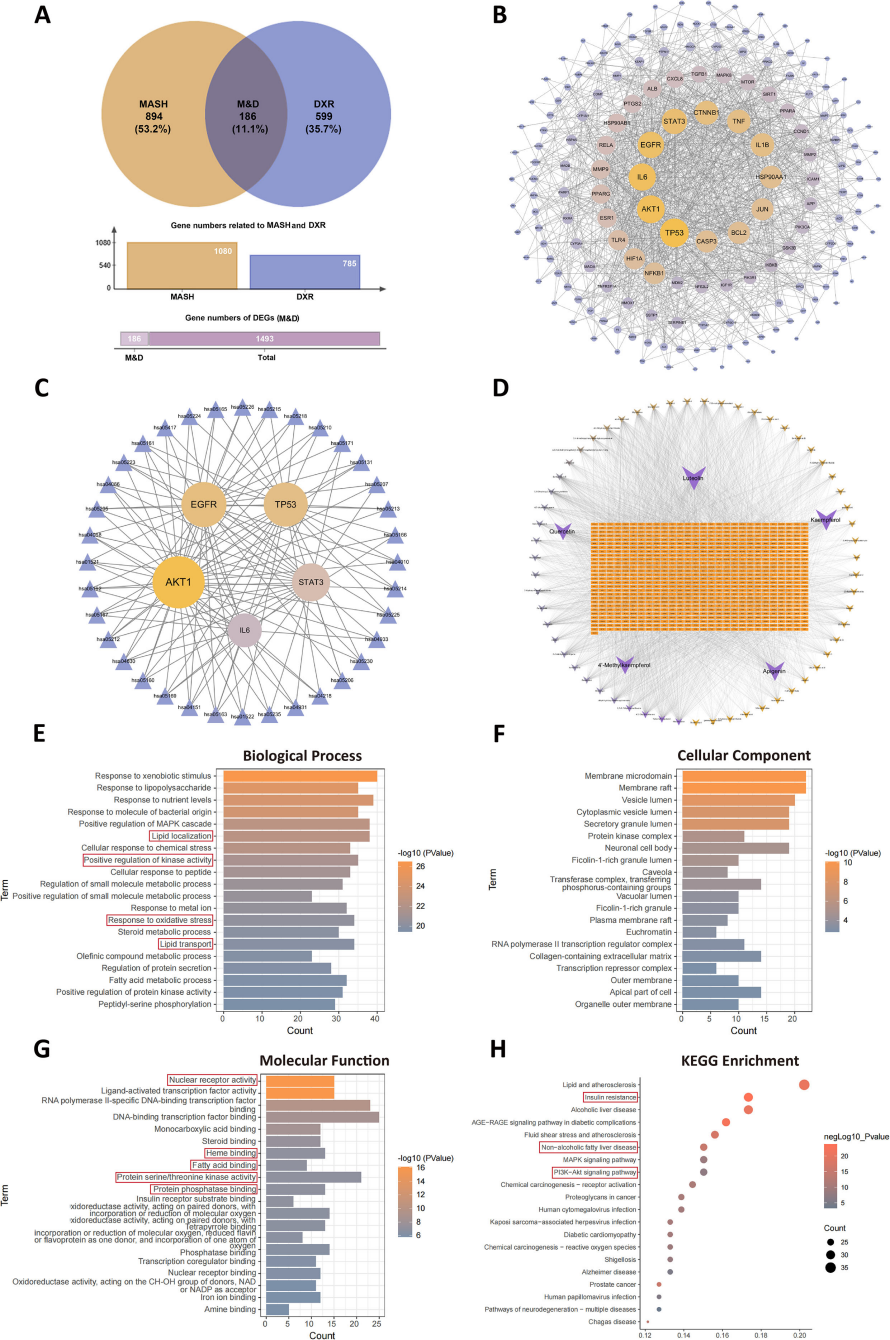
Bioinformatic analysis of DXR targets in MASH. **(A)** Core gene hub (M&D) identified by intersecting MASH-related genes and DXR targets. **(B–D)** PPI network, target-pathway network, and component-target network highlighting hub genes and major active compounds. **(E–G)** GO enrichment showing lipid metabolism, oxidative stress, and kinase activity-related processes. **(H)** KEGG enrichment showing PI3K/AKT signaling, insulin resistance, and NAFLD pathways.

A total of 1080 pathological genes related to MASH were obtained from the GeneCards and OMIM databases (**Figure 4A**). Utilizing the SwissTargetPrediction database, 785 targets of the main active components derived from DXR were predicted. The core gene hub of DXR in the treatment of MASH (M&D), comprising 186 genes, was established by intersecting the pathological genes and the predicted targets (**Figure 4A**, **Supplementary Table 5**). The herb-constitute-target interaction network was then constructed. Among the 8 herbs in DXR, GL, MA, AM, PC, LL, PM, LC, and AS contained 30, 28, 62, 4, 35, 12, 33, and 4 active components, respectively (**Supplementary Figure 1**).

Focusing on the targets of DXR in the treatment of MASH, comprehensive bioinformatic analyses were employed. Firstly, a protein-protein interaction network was constructed using STRING. Topological analysis revealed that TP53, AKT1, IL6, EGFR, STAT3 exhibited the most significant top 5 degrees in the M&D (**Figure 4B**). Subsequently, a target-pathway interaction network was constructed, with topological analysis highlighting AKT1, EGFR, TP53, STAT3, and IL6 as the top 5 genes in M&D (**Figure 4C**). The constituent-target interaction network indicated that the top 5 active components derived from DXR were quercetin, kaempferol, apigenin, luteolin, and 4’-Methoxykampferol (**Figure 4D**).

Moreover, GO enrichment analysis demonstrated that in the biological process, terms such as “Lipid localization”, “Positive regulation of kinase activity”, “Response to oxidative stress”, and “Lipid transport” ranked among the top 15, closely related to lipid metabolism, oxidative stress and kinase activity (**Figure 4E**). In the cellular component, “Membrane microdomain”, “Membrane raft”, “Vesicle lumen”, “Cytoplasmic vesicle lumen”, and “Secretory granule lumen” ranked among the top 5, indicating that the pathological regions were mainly membrane and cytoplasm (**Figure 4F**). In the molecular function, “Nuclear receptor activity”, “Heme binding”, “Fatty acid binding”, “Protein serine/threonine kinase activity”, and “Protein phosphatase binding” ranked among top 10, closely associated with nuclear receptor signaling and protein kinase phosphorylation (**Figure 4G**). KEGG pathway analysis also indicated that the “Insulin resistance” ranked 2nd, “Non-alcoholic fatty liver disease” ranked 6th, and “PI3K/AKT signaling pathway” ranked 8th (**Figure 4H**). These results suggest that the predicted targets of DXR are primarily associated with biological processes related to lipid metabolism, oxidative stress, and kinase regulation. Among the enriched pathways, PI3K/AKT signaling and nuclear receptor–related pathways emerged as prominently represented candidates, providing a rationale for their prioritization in subsequent experimental validation rather than establishing direct causal involvement at this stage.

### 3.4 DXR suppresses lipid synthesis, inflammatory response, and oxidative stress in diet-induced MASH mice

**Figure 5:**
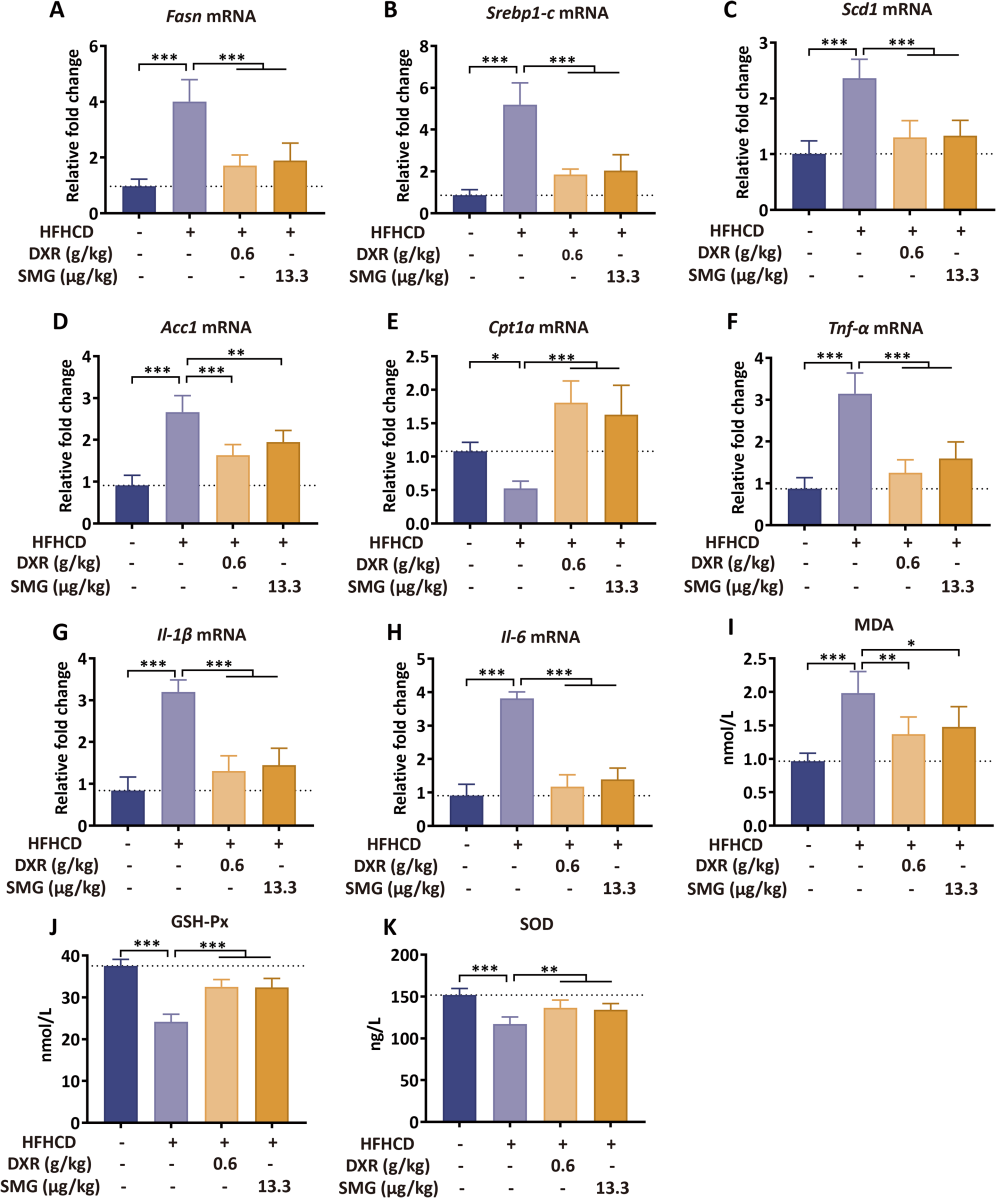
DXR modulates lipid synthesis, inflammation, and oxidative stress in liver. **(A–E)** DXR downregulated Fasn, Srebp1-c, Scd1, Acc1 and upregulated Cpt1a. **(F–H)** DXR reduced Tnf-α, Il-1β, and Il-6 mRNA levels. **(I–K)** DXR decreased MDA and increased SOD and GSH-Px. ^*^P < 0.05; ^**^P < 0.01; ^***^P < 0.001.

Due to the pathological processes of MASH, the accumulation of hepatic lipids and inflammatory responses are two major manifestations. We further observed the mRNA expression patterns related to hepatic lipid synthesis and inflammatory reactions. The results indicate that DXR and SMG downregulated mRNA expressions of *Fasn*, *Srebp1-c*, *Scd1*, and *Acc1*, while upregulating the expression of *Cpt1a*, thereby inhibiting lipid synthesis and regulating lipid homeostasis (**Figures 5A–E**). Furthermore, DXR and SMG decreased mRNA expressions of *Tnf-α*, *Il-1β*, and *Il-6*, ameliorating hepatic inflammation (**Figures 5F–H**). Additionally, DXR and SMG reduced hepatic MDA levels, increased SOD, and elevated GSH-Px compared to the HFHCD group, thus mitigating oxidative stress in MASH mice induced by HFHCD (**Figures 5I–K**). These findings suggest that DXR alleviates MASH by inhibiting lipid synthesis, inflammatory reactions, and oxidative stress.

### 3.5 Hepatoprotective and anti-apoptotic effects of DXR

Several studies have indicated that excessive oxidative stress can induce cell apoptosis, ultimately leading to liver injury and fibrosis in MASH (18, 19). To evaluate the apoptosis of hepatocytes after DXR treatment, we conducted experiments including TUNEL staining and IHC staining of Bax and Bcl-2 in liver tissues.

**Figure 6:**
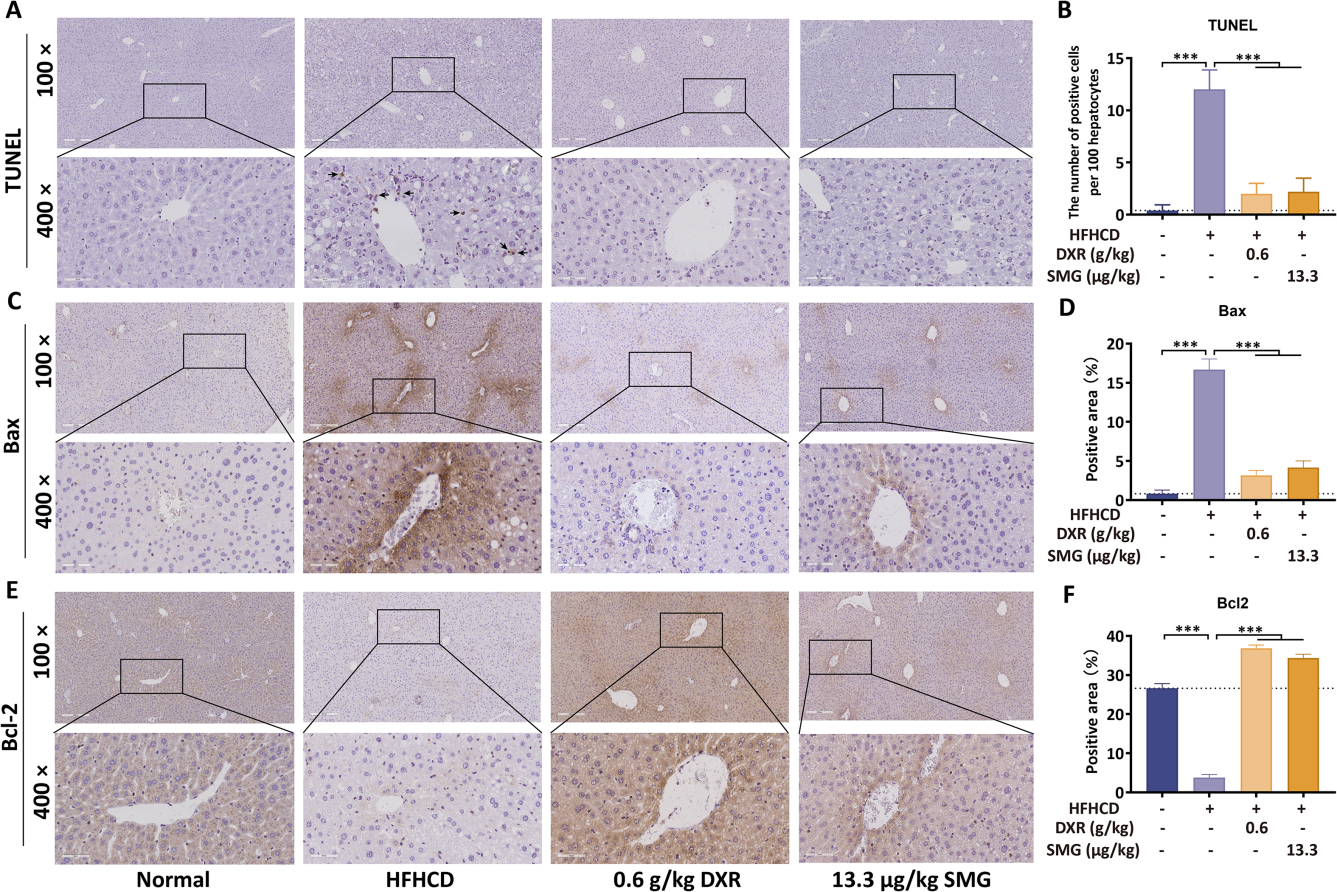
DXR protects hepatocytes from apoptosis. **(A, B)** TUNEL staining showed reduced apoptotic cells after DXR treatment (scale bar = 200 μm or 50 μm). **(C–F)** Western blot and IHC revealed decreased Bax and increased Bcl-2 expression. ^***^P < 0.001 vs. HFHCD.

The number of apoptotic hepatocytes significantly increased in the HFHCD group compared with the normal group, but apoptotic cells markedly decreased after treatment with DXR or SMG (**Figures 6A, B**). The black arrows marked the apoptotic cells, which exhibited cell shrinkage and nuclear pyknosis. The expression of Bax increased in mice fed the HFHC diet, but it significantly decreased in mice treated with DXR or SMG (**Figures 6C, D**). Conversely, the expression of Bcl-2 showed the opposite trend. It prominently reduced after treatment with the HFHC diet, but increased in the DXR and SMG groups (**Figures 6E, F**). These results strongly support the protective effects of DXR against hepatocyte apoptosis, ameliorating liver injury.

### 3.6 DXR activates PI3K/AKT and Keap1/Nrf2 signaling *in vivo*

**Figure 7:**
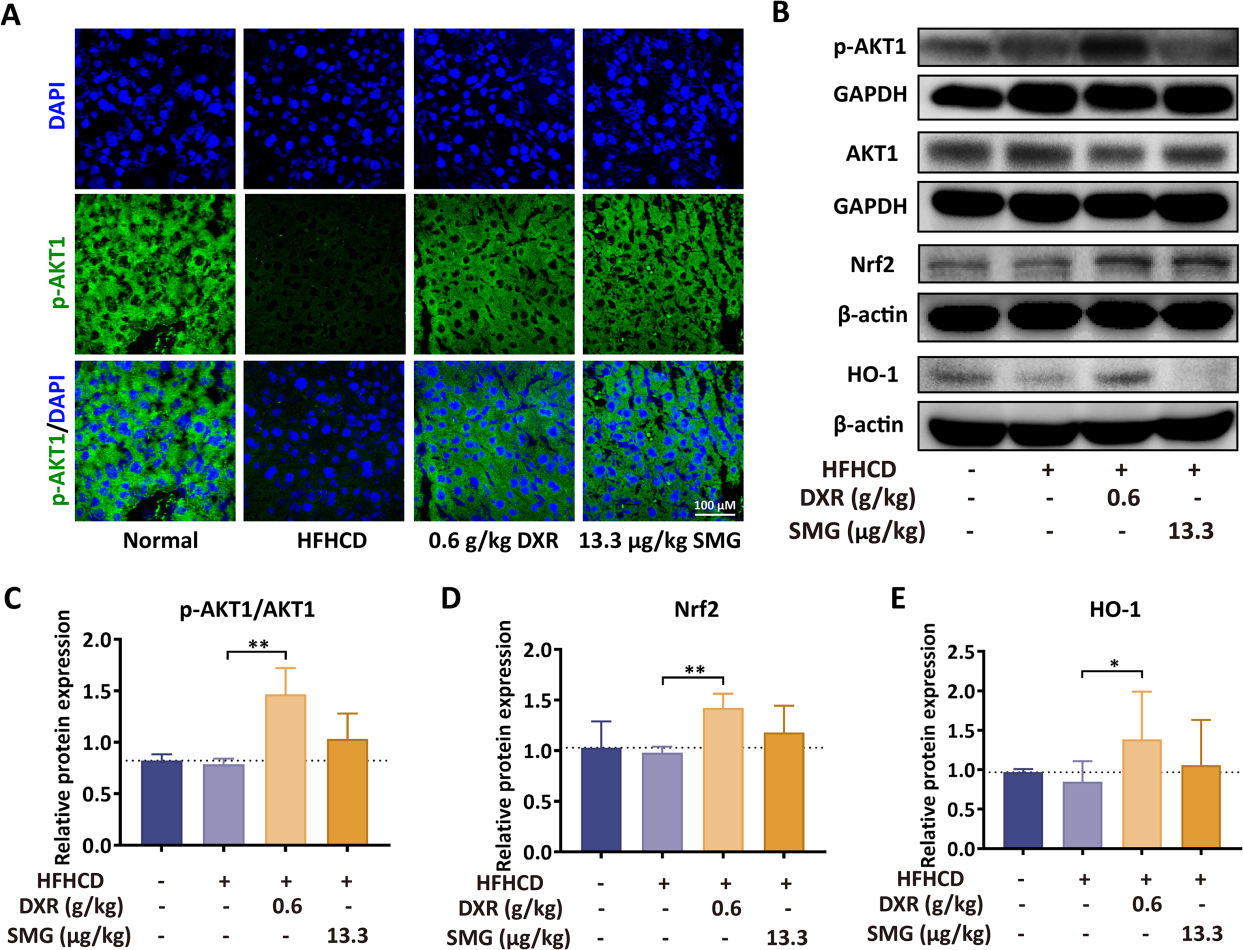
DXR activates PI3K/AKT and Keap1/Nrf2/HO-1 signaling. **(A)** Immunofluorescence showed increased p-AKT1 after DXR treatment (scale bar = 100 μm). **(B–E)** Western blot and quantification demonstrated upregulation of p-AKT1, Nrf2, and HO-1. ^*^P < 0.05; ^**^P < 0.01; ^***^P < 0.001.

Bioinformatic analysis results suggested that the core signaling pathway of DXR in the treatment of MASH is PI3K/AKT. To verify this hypothesis, the phosphorylation of AKT1 in liver tissues was detected by immunofluorescence and Western Blotting. In the cytoplasm of hepatocytes, the fluorescence density of phosphorylated-AKT1 (green) decreased in the HFHCD group, and it increased after treatment with DXR and SMG (**Figure 7A**). The ratio of phosphorylated-AKT1 to total AKT1 also decreased in the HFHCD group and increased after treatment with DXR and SMG (**Figures 7B, C**).

Next, we further examined changes in the classical oxidative stress signaling pathway Keap1/Nrf2/HO-1. Compared to the normal control group, hepatic Nrf2 and HO-1 protein expression in the HFHCD group showed a slight decrease or remained similar, whereas both proteins were significantly upregulated following DXR treatment (**Figures 7B, D, E**).

We then evaluated the mRNA expression levels of a series of classical Nrf2 downstream target genes in mouse liver tissue (**Supplementary Figure 3**), including Nqo1, Gclc, Gclm, Srxn1, and Gsta4. The results demonstrated that DXR treatment significantly upregulated the mRNA expression of Nqo1, Gclc, and Gclm compared to the HFHCD group. Notably, Srxn1 and Gsta4 exhibited a certain degree of elevation in the HFHCD group, with their expression further enhanced following DXR intervention.

These findings collectively support the activating effect of DXR on the Keap1/Nrf2 antioxidant pathway at both the protein and transcriptional levels, suggesting that DXR may alleviate hepatic lipid accumulation, inflammatory responses, and oxidative damage through upregulation of antioxidant defense-related gene expression. Furthermore, combined with the concurrent activation of the PI3K/AKT pathway, these results indicate that DXR may exert its protective effects against MASH through coordinated regulation of multiple signaling pathways.

### 3.7 Exploration of candidate DXR constituents potentially interacting with PI3K and Keap1 by *in silico* analysis

To further elucidate the regulatory effects of key active components in DXR on PI3K and Keap1, we employed virtual molecular docking and molecular dynamics simulation. We observed the binding energies of the top 4 flavonoid components, quercetin, kaempferol, apigenin, and luteolin, with PI3K and Keap1. Quercetin, luteolin, kaempferol, and apigenin obtained binding scores with PI3K of -9.042, -8.976, -8.201, and -7.605 kcal/mol, respectively. Similarly, with Keap1, their binding scores were -8.474, -8.679, -8.169, and -8.552 kcal/mol (**Table 1**). The 3D structures of the top 4 flavonoids binding with PI3K and Keap1 indicated that the three molecules are right docked into the catalytic pocket of PI3K and Keap1 (**Supplementary Figure 4**). As the 2D diagrams of flavonoids-PI3K show, Lys833, Val882, and Asp964 were the key amino acid residues that form the hydrogen bond with the 7-hydroxyl, 2-(4-hydroxyphenyl), and 4-carbonyl of both four flavonoids. Asp836 can form hydrogen bonds with the 5-hydroxy group in quercetin, apigenin, and luteolin (**Supplementary Figures 4A–D**). In kaempferol, the 7-hydroxy group can simultaneously form hydrogen bonds with Ser806 and Lys833. As the 2D diagrams of flavonoids-Keap1 show, The key amino acid residue Leu365 can form hydrogen bonds with the 5-hydroxy group of apigenin, kaempferol, and luteolin (**Supplementary Figures 3E–H**). Simultaneously, the amino acid side chains Ser508-Val512 play a crucial role in the binding of flavonoids to Keap1. Several amino acid residues within this region, such as Ser508 and Ala510, can form hydrogen bonds with apigenin, luteolin, and kaempferol (**Supplementary Figures 4F–H**).

**Table 1:** Active components derived from DXP docking into PI3K and Keap1.

Component	PI3K (kcal/mol)	Keap1 (kcal/mol)
Quercetin	-9.042	-8.474
Luteolin	-8.976	-8.679
Kaempferol	-8.201	-8.169
Apigenin	-7.605	-8.552

**Figure 8:**
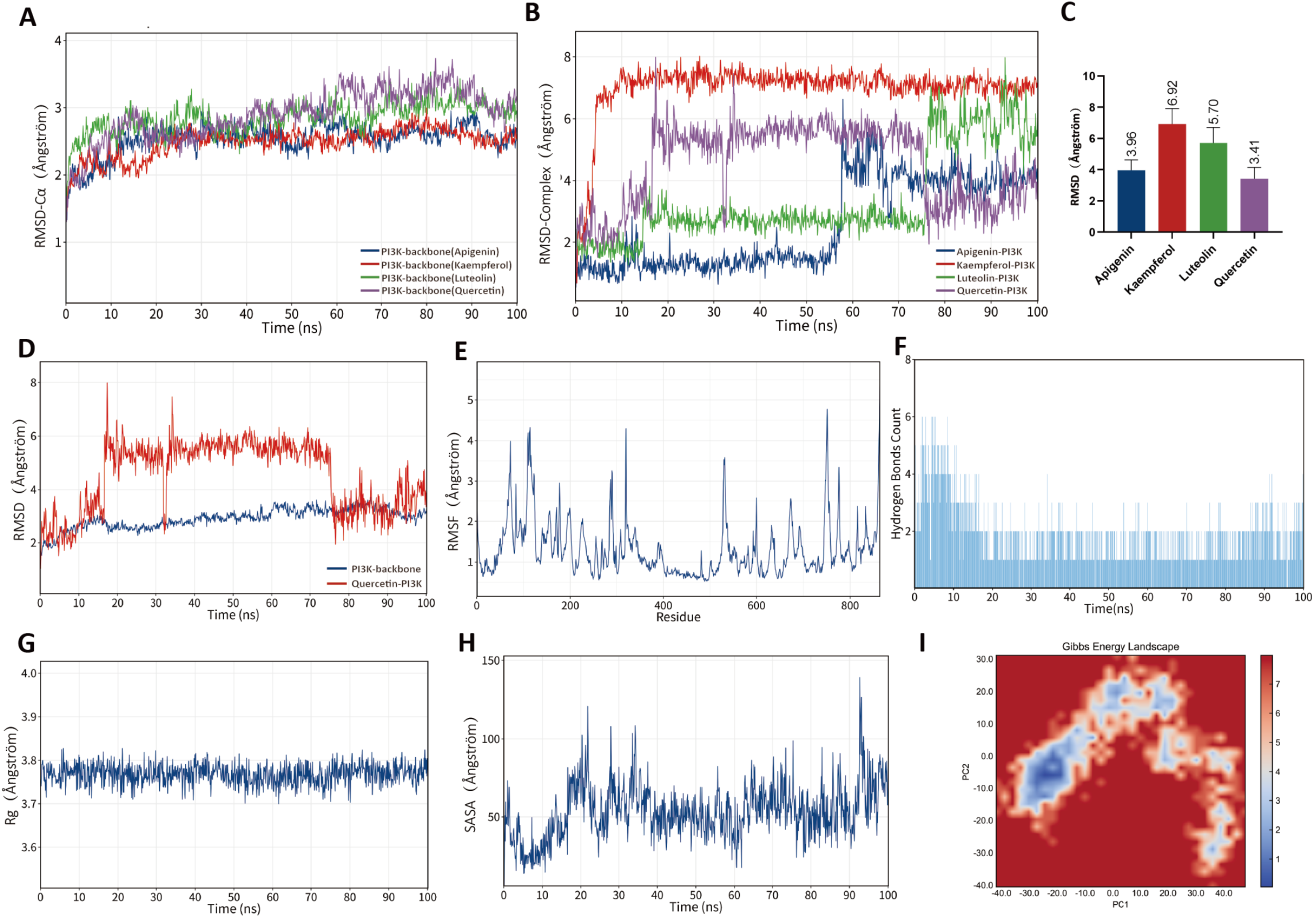
Molecular dynamics of DXR flavonoids with PI3K. **(A–I)** RMSD, RMSF, hydrogen bonds, Rg, SASA, and FEL analyses showed stable binding of quercetin, luteolin, kaempferol, and apigenin with PI3K.

**Figure 9:**
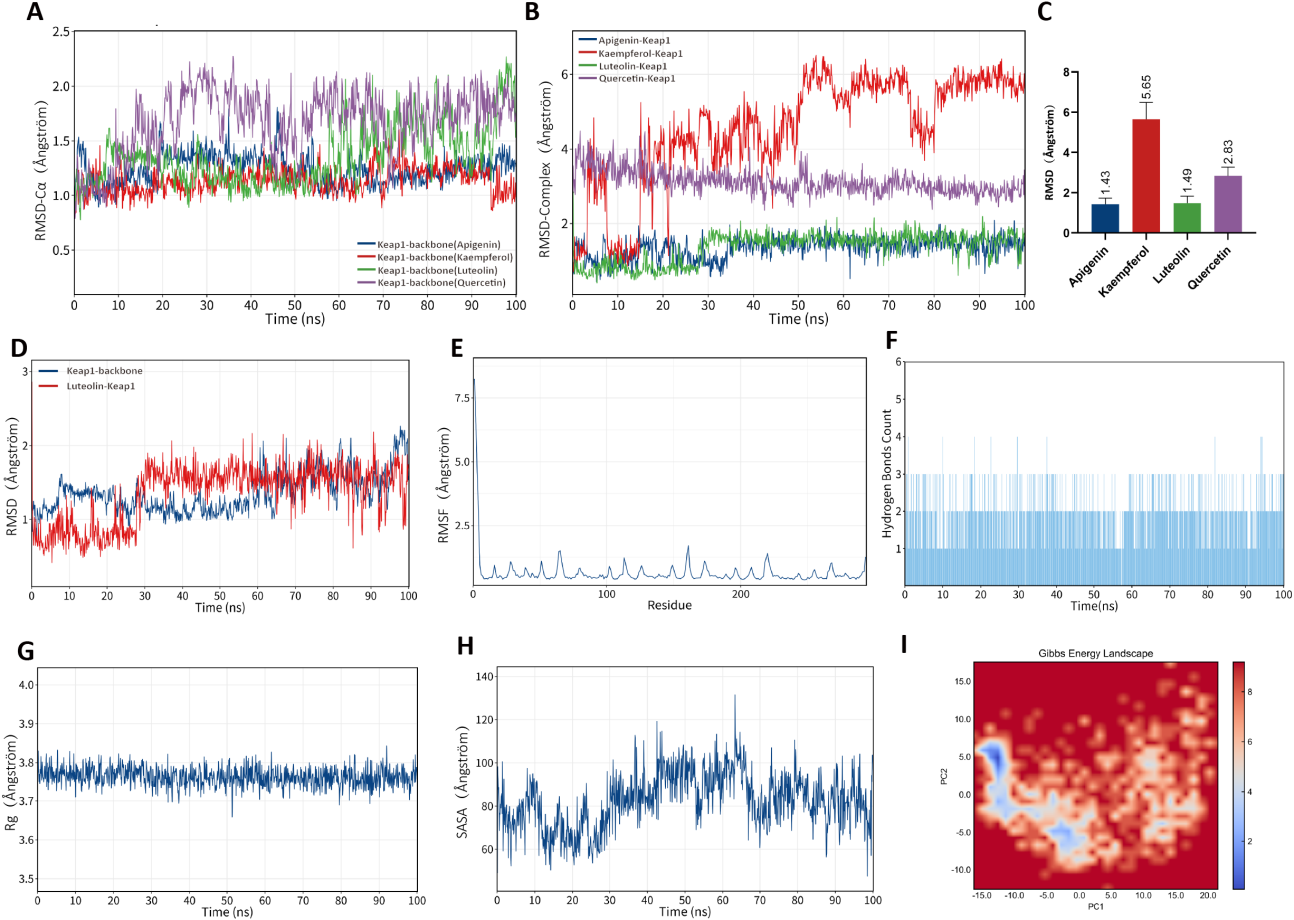
Molecular dynamics of DXR flavonoids with Keap1. **(A–I)** RMSD, RMSF, hydrogen bonds, Rg, SASA, and FEL analyses indicated stable binding of quercetin, luteolin, kaempferol, and apigenin with Keap1.

Molecular dynamics (MD) simulation results showed that the four flavonoids (quercetin, luteolin, kaempferol, and apigenin) were able to maintain structural stability after forming complexes with Keap1 and PI3K proteins. Backbone RMSD analysis (**Figures 8A**, **9A**) indicated that the overall fluctuations of the systems remained small throughout the simulation, demonstrating high stability. Further complex RMSD analysis (**Figures 8B**, **9B**) revealed that the Apigenin–Keap1 and Quercetin–PI3K complexes exhibited the lowest RMSD values with minimal fluctuations, suggesting superior binding stability compared with the other flavonoids. Therefore, subsequent analyses focused on the Apigenin–Keap1 and Quercetin–PI3K complexes.

RMSF analysis (**Figures 8D**, **9D**) showed that the overall residue flexibility was low, with only slight fluctuations observed in some loop regions, indicating that the protein structures remained stable upon ligand binding. Moreover, Rg analysis (**Figures 8F**, **9F**) demonstrated that the complexes maintained relatively stable radius values throughout the simulations, suggesting no significant change in structural compactness. SASA analysis (**Figures 8G**, **9G**) showed stable solvent-accessible surface areas, implying that no obvious exposure or collapse occurred. Hydrogen bond analysis (**Figures 8H**, **9H**) further revealed the formation of persistent and stable hydrogen bonds between ligands and their respective proteins, which contributed critically to complex stability. Finally, free energy landscape (FEL) analysis (**Figures 8I**, **9I**) demonstrated that both the Apigenin–Keap1 and Quercetin–PI3K complexes were characterized by relatively concentrated and low-energy stable conformational clusters, further supporting their thermodynamic stability. Molecular docking combined with molecular dynamics simulations demonstrate that these flavonoid compounds exhibit favorable structural compatibility and stable binding modes with PI3K and Keap1. These *in silico* simulation results provide theoretical and structural basis for the potential modulation of PI3K/AKT and Keap1/Nrf2 pathways by DXR-derived flavonoids. Among these, quercetin with PI3K and apigenin with Keap1 displayed relatively more stable interaction patterns, suggesting that they may serve as priority candidate compounds for further experimental validation, rather than constituting direct evidence of definitive physical binding or functional regulatory activity.

### 3.8 The effects of four flavonoids on the regulation of p-AKT1 and Nrf2 *in vitro*

**Figure 10:**
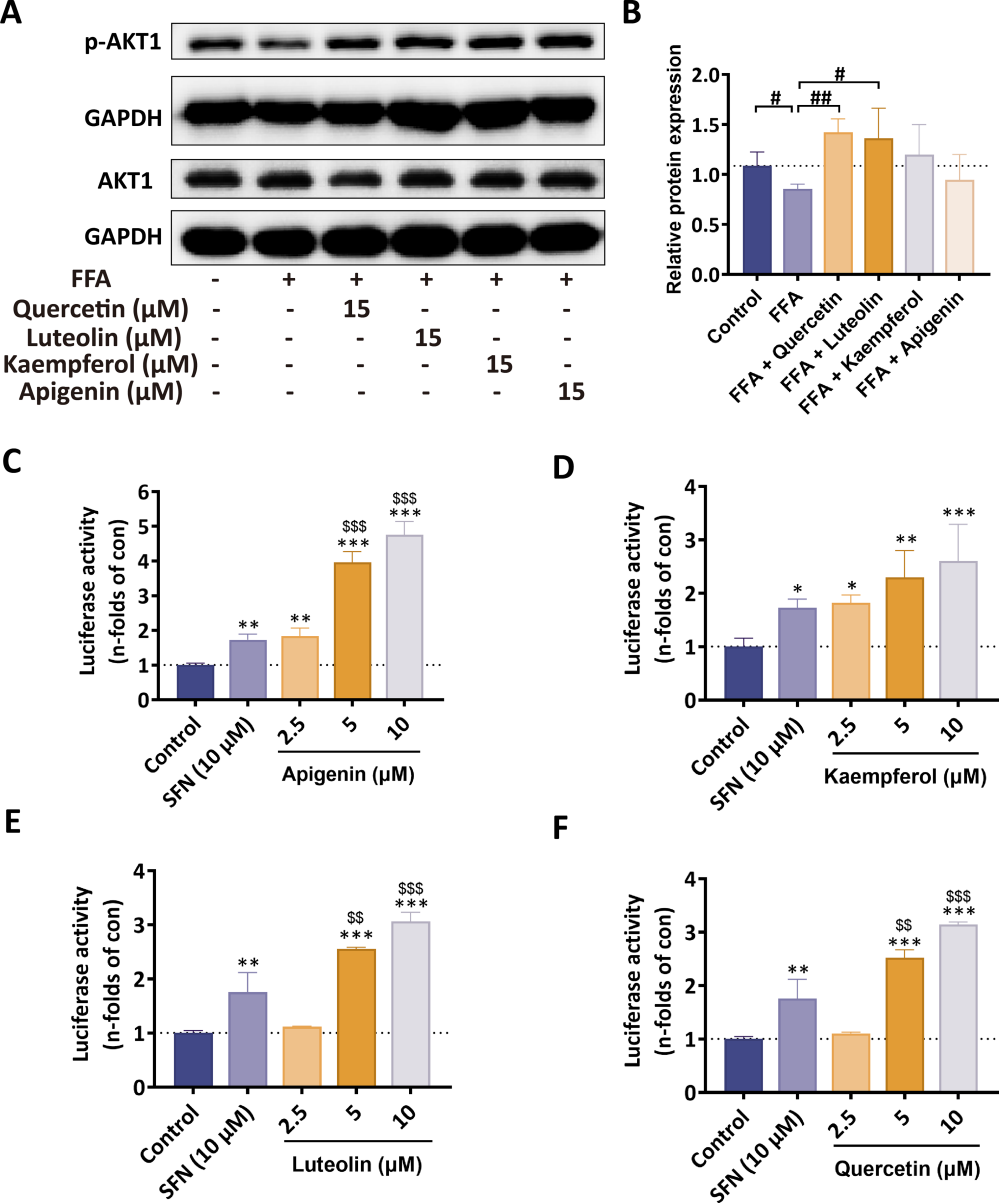
Four flavonoids regulate p-AKT1 and Nrf2 *in vitro*. **(A, B)** Quercetin and luteolin increased p-AKT1 levels in AML12 cells, with quercetin showing a stronger effect. **(C–F)** Quercetin, kaempferol, apigenin, and luteolin activated Nrf2 luciferase activity in HEK293-Nrf2-luc cells, with apigenin exhibiting the strongest activation. ^#^P < 0.05; ^##^P < 0.01; ^###^P < 0.001 vs. HFHCD; ^*^P < 0.05; ^**^P < 0.01; ^***^P < 0.001 vs. control; ^$$^P < 0.01; ^$$$^P < 0.001 vs. SFN.

Subsequently, we established an *in vitro* hepatosteatosis model using AML12 cells by intervening with free fatty acids (FFA), a mixture of oleic acid and palmitic acid (2: 1). Based on this, we separately administered four flavonoid compounds for intervention. Since previous experiments have observed the therapeutic effects of these four flavonoids on FFA-induced normal liver cells, a concentration of 15 μM was used for observation in this experiment (20). The results showed that all four flavonoid compounds could increase the protein expression levels of phosphorylated AKT1 to varying degrees. Among them, the increase in quercetin seemed to be the most prominent, but no significant statistical differences were observed compared to the other three compounds (**Figures 10A, B**).

To further evaluate Nrf2 transcriptional activity, a luciferase reporter assay was performed using the HEK293-Nrf2-luc cell line. After co-incubating with different concentrations of the four flavonoid compounds for 24 hours, it can be observed that, compared to the control group, the fluorescence intensity of all four compounds increased to varying degrees at the concentration of 15 μM (**Figure 10C**). Among them, apigenin exhibited the most potent Nrf2 agonistic effect compared to the other three tested compounds, while the Nrf2 agonistic effects of other three compounds were relatively weak (**Figure 10D**). The reporter assay provided supportive functional evidence of Nrf2 activation, and the results were consistent with the molecular dynamics simulation results. Combining our previous observations of the regulatory effects of these four flavonoids on reducing lipid accumulation in AML12 cells induced by FFA (20), this further demonstrates that these four flavonoid compounds can exert their anti-MASH effects by upregulating the Keap1/Nrf2 signaling pathway, with apigenin showing the most prominent effect.

## 4 Discussion

MASH represents a critical stage in the progression of MASLD, with its pathogenesis involving lipid deposition, inflammatory responses, oxidative stress, and fibrosis (21, 22). In recent years, the pursuit of effective MASH therapies through multi-target and multi-pathway interventions has emerged as an important research frontier in hepatology (23). Traditional Chinese medicine formulae, characterized by their multi-component and multi-target properties, have shown unique advantages in the treatment of MASH (24). The present study revealed the protective role of DXR in MASH, particularly through modulation of the PI3K/AKT and Keap1/Nrf2 signaling pathways, suggesting its potential as a candidate drug with translational promise. This finding not only validates the rationale of TCM formulae in modern disease management but also provides new insights into the molecular pathogenesis of MASH.

From the perspective of pathological mechanisms, the dynamic balance between lipid synthesis and breakdown is central to hepatic metabolic homeostasis. In the MASH state, upregulation of lipogenic genes such as Fasn, Srebp1-c, and Scd1 exacerbates hepatocellular lipid accumulation, leading to abnormal membrane tension and endoplasmic reticulum stress, which subsequently trigger inflammation and apoptosis (25). DXR was shown to downregulate these genes while upregulating Cpt1a, thereby suppressing *de novo* lipogenesis and promoting fatty acid oxidation at the “source.” This mode of action is reminiscent of the mechanism of PPARα agonists in clinical use, yet the synergistic effects of multiple components in the formula confer a broader regulatory spectrum (26). Importantly, such comprehensive regulation of lipid metabolism pathways may provide more robust metabolic improvements for the long-term management of MASH.

Inflammatory responses are a key driver of MASH progression, originating not only from the activation of Kupffer cells and macrophages but also from hepatocyte-derived inflammatory mediators (27, 28). The upregulation of cytokines such as TNF-α, IL-1β, and IL-6 exacerbates local hepatic injury and may also impair systemic insulin sensitivity, perpetuating a vicious cycle of metabolic inflammation (29). Our study demonstrated that DXR significantly suppressed the expression of these cytokines, suggesting its potential to modulate inflammasome activity and NF-κB signaling, thereby ameliorating the pro-inflammatory milieu associated with MASH. This effect is particularly critical for halting the transition from lipotoxicity to inflammatory injury.

Oxidative stress occupies a central role in MASH pathophysiology. Excessive reactive oxygen species (ROS) production combined with insufficient antioxidant defenses leads to lipid peroxidation, mitochondrial injury, and apoptosis (30, 31). The Keap1/Nrf2 pathway, a canonical endogenous antioxidant defense mechanism, directly determines the capacity of cells to eliminate ROS (32). DXR markedly upregulated Nrf2 and HO-1 expression, indicating its ability to relieve Keap1-mediated repression of Nrf2 and thereby enhance antioxidant defenses. This not only attenuates lipid peroxidation-induced hepatocellular damage but may also improve mitochondrial dysfunction, ultimately stabilizing hepatic metabolic homeostasis. Of note, antioxidative effects in MASH often intersect with anti-inflammatory and anti-fibrotic mechanisms; thus, DXR-mediated regulation of Nrf2 signaling may confer multilayered protective benefits.

From a pathway-level perspective, KEGG enrichment analysis also identified several other pathways potentially relevant to MASH, which are reported in the Results but were not explored in depth due to scope and feasibility considerations rather than selective reporting. Among these pathways, PI3K/AKT signaling was prioritized based on its established roles in metabolic regulation and its consistency with the phenotypic and molecular changes observed in the HFHCD model.

Building on this mechanistic framework, another highlight of this study lies in the identification of bioactive compounds within the formula and the elucidation of their molecular mechanisms. Flavonoids such as quercetin, apigenin, kaempferol, and luteolin exhibited favorable affinities for PI3K and Keap1 in molecular docking and dynamics simulations, and their regulatory effects on p-AKT1 and Nrf2 were further validated *in vitro*. Notably, the flavonoids displayed distinct pathway preferences: quercetin showed stronger stability in PI3K/AKT signaling, whereas apigenin demonstrated greater efficacy in activating Keap1/Nrf2. This “homologous molecules–heterogeneous functions” feature may underlie the ability of multi-component formulae to achieve multi-layered regulation in complex pathological states, further emphasizing the rationale and necessity of TCM-based multi-component interventions.

From a signaling perspective, dual regulation of PI3K/AKT and Keap1/Nrf2 pathways is of particular importance. PI3K/AKT is not only a critical regulator of metabolic homeostasis but also a central node in anti-apoptotic and anti-inflammatory responses (33). Its impairment is often associated with insulin resistance, lipid accumulation, and cell death (34). Meanwhile, the Nrf2 pathway, as the core of cellular antioxidant and detoxification systems, enables defense against both exogenous and endogenous stresses (35). These two pathways interact during MASH progression: activation of PI3K/AKT may indirectly enhance Nrf2 transcriptional activity, while Nrf2 upregulation contributes to alleviating oxidative stress, thereby stabilizing AKT signaling. The ability of DXR to modulate both pathways suggests an integrated mechanism by which metabolic, inflammatory, and oxidative stress processes are collectively regulated, aligning well with the multifaceted pathology of MASH. Nevertheless, although multiple downstream targets and reporter assays support Nrf2 activation, direct evidence of Nrf2 nuclear translocation was not assessed, and the HEK293-based reporter system used in this study does not fully recapitulate hepatocyte-specific regulation in the pathological context of MASH, both of which warrant further investigation.

An important consideration is whether the pronounced efficacy of DXR observed in animal models can be translated into sustained clinical benefits. Patients with MASH frequently present with comorbidities such as obesity, type 2 diabetes, and dyslipidemia, conditions in which monotherapy targeting a single pathway often proves insufficient (36). By exerting comprehensive effects through multiple pathways and bioactive constituents, DXR theoretically aligns more closely with the therapeutic needs of such complex metabolic disorders. Furthermore, flavonoids possess a well-established safety and tolerability profile supported by preclinical and clinical data, providing a favorable basis for translational studies (37, 38). Nevertheless, challenges remain regarding dose standardization, compositional consistency, and clarification of pharmacologically active constituents, which will ultimately determine whether DXR can be incorporated into the framework of evidence-based medicine (39).

## 5 Conclusion

In summary, DXR exerts potent therapeutic effects against MASH by ameliorating hepatic steatosis, inflammation, and fibrosis in both dietary-induced and MCD-induced murine models. Mechanistically, DXR suppresses hepatic lipid synthesis and accumulation by down-regulating key lipogenic enzymes (FASN, SCD1, and ACC1) while enhancing fatty acid oxidation through the up-regulation of CPT1a. Moreover, DXR markedly attenuates oxidative stress and inflammation, as evidenced by decreased levels of MDA, TNF-α, IL-1β, and IL-6, and enhanced antioxidant enzyme activities (SOD and GSH-Px). It also protects hepatocytes from apoptosis by up-regulating Bcl-2 and down-regulating Bax expression.

At the molecular level, DXR activates both the PI3K/AKT and Keap1/Nrf2 signaling pathways, which play pivotal roles in mediating its hepatoprotective effects. Phytochemical profiling and molecular dynamics simulations identify four major flavonoids (apigenin, kaempferol, luteolin, and quercetin) as key bioactive constituents. Among them, quercetin promotes AKT1 phosphorylation to activate the PI3K/AKT pathway, whereas all four flavonoids enhance Nrf2 activation, with apigenin demonstrating the strongest effect.

Collectively, these findings highlight that DXR alleviates MASH through a multifaceted mechanism involving lipid metabolism regulation, antioxidant defense, anti-inflammatory response, and anti-apoptotic activity. As a promising natural formula targeting both the PI3K/AKT and Keap1/Nrf2 pathways, DXR represents a potential novel therapeutic candidate for the prevention and treatment of MASH, warranting further pharmacological and clinical investigation.

## Data Availability

The raw data supporting the conclusions of this article will be made available by the authors, without undue reservation.

## Supplementary material

Supplementary Figure 1 The fingerprint profile of DXR detected by UHPLC-Q-Orbitrap HRMS. ESI+ represents the positive ion mode, and ESI- represents the negative ion mode.

Supplementary Figure 2 The herb-component-target network and topological analysis. The active components of DXR were detected by UHPLC-Q-Orbitrap HRMS and their predicted targets were collected from SwissTargetPrediction database. Then, the topological analysis was performed using Cytoscape software. The predicted targets were signed with orange boxes. The herbs were signed with dark green rhombuses and their active components were signed with light green circles. LC (*Ligusticum chuanxiong*), MA (*Morus alba*), GL (*Ganoderma lucidum*), AM (*Astragalus membranaceus*), LL (*Ligustrum lucidum*), PC (*Poria cocos*), and PM (*Prunus mume*).

Supplementary Figure 3 DXR upregulates hepatic Nrf2 downstream antioxidant gene expression in HFHCD-fed mice. Relative mRNA expression levels of Nqo1 **(A)**, Gclc **(B)**, Gclm **(C)**, Srxn1 **(D)**, and Gsta4 **(E)** in mouse liver tissues were measured by qRT-PCR. Compared with the HFHCD group, DXR significantly increased the mRNA levels of Nqo1, Gclc, and Gclm. Srxn1 and Gsta4 were elevated in the HFHCD group and were further upregulated after DXR treatment. ^*^P < 0.05; ^**^P < 0.01; ^***^P < 0.001 vs. HFHCD.

Supplementary Figure 4 The virtual molecular docking of PI3K and top 4 active components derived from DXR. The 3D docking illustration, 3D and 2D interaction diagrams of PI3K (PDB ID: 5JHA) with quercetin **(A)**, luteolin **(B)**, kaempferol **(C)**, apigenin **(D)**, respectively. The 3D docking illustration, 3D and 2D interaction diagrams of Keap1 (PDB ID: 8IVG) with quercetin **(E)**, luteolin **(F)**, kaempferol **(G)**, apigenin **(H)**, respectively. The yellow dotted line (middle) and purple arrow (right) represent the hydrogen bonds.
