# Supplementary figures and images for: Daixie recipe ameliorates diet-induced MASH in mice via activating PI3K/AKT and Keap1/Nrf2 signaling
Source: Front Endocrinol (Lausanne). 2026 Mar 13;17:1772033. doi: 10.3389/fendo.2026.1772033 (PMC13021464; doi:10.3389/fendo.2026.1772033)

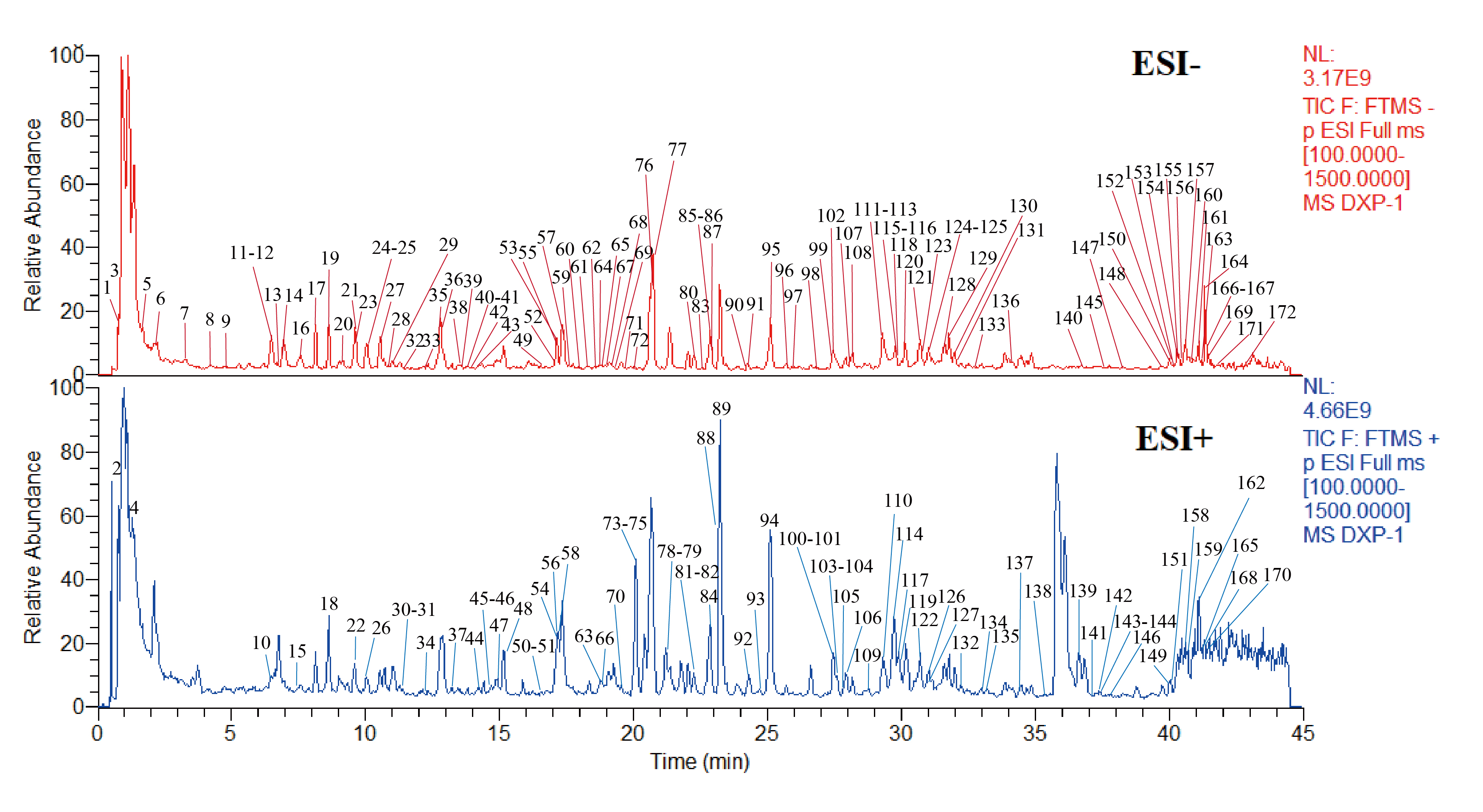

Supplement: Supplementary Figure 1 — The fingerprint profile of DXR detected by UHPLC-Q-Orbitrap HRMS. ESI+ represents the positive ion mode, and ESI- represents the negative ion mode. [file Image1.jpeg]

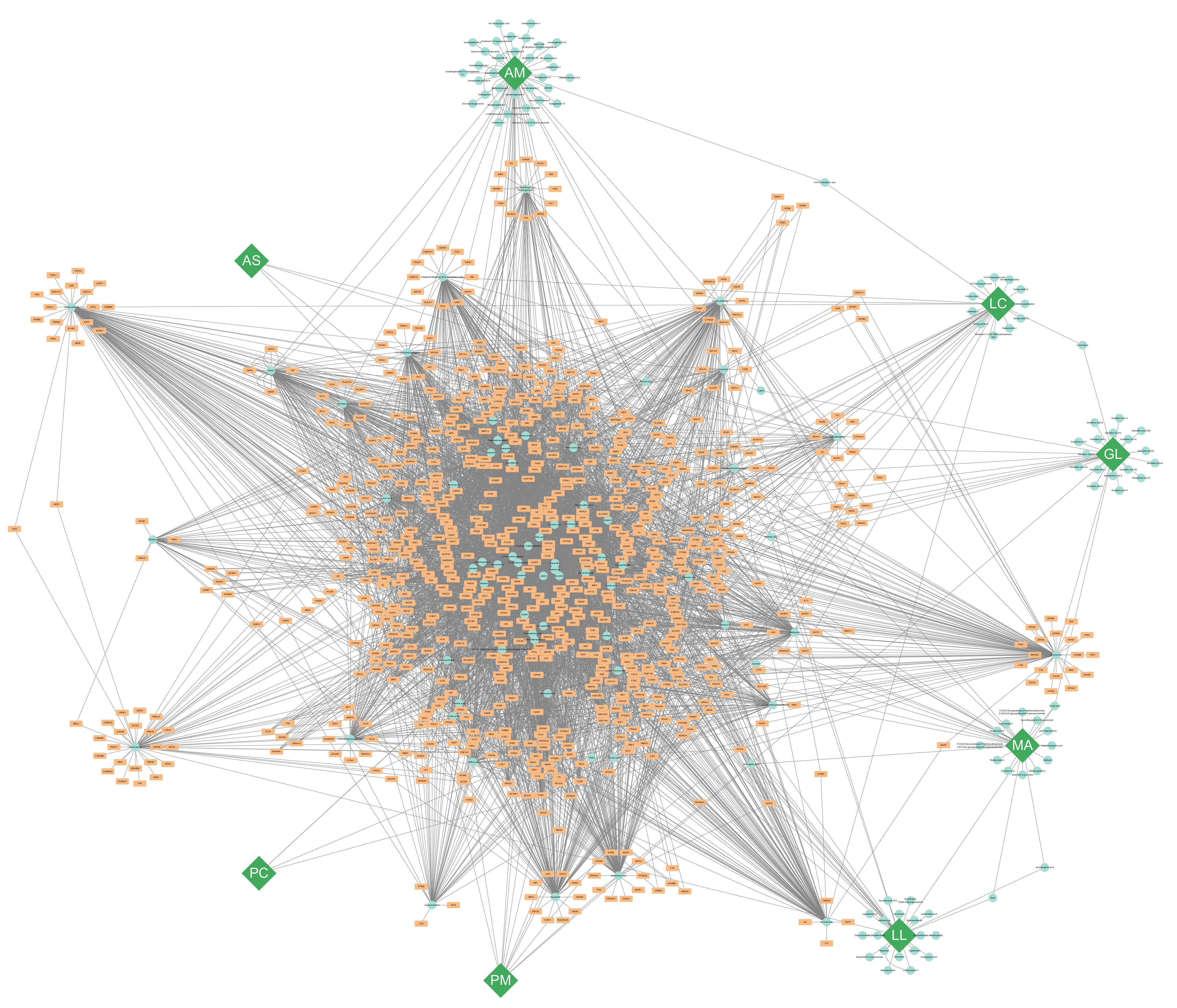

Supplement: Supplementary Figure 2 — The herb-component-target network and topological analysis. The active components of DXR were detected by UHPLC-Q-Orbitrap HRMS and their predicted targets were collected from SwissTargetPrediction database. Then, the topological analysis was performed using Cytoscape software. The predicted targets were signed with orange boxes. The herbs were signed with dark green rhombuses and their active components were signed with light green circles. LC (Ligusticum chuanxiong), MA (Morus alba), GL (Ganoderma lucidum), AM (Astragalus membranaceus), LL (Ligustrum lucidum), PC (Poria cocos), and PM (Prunus mume). [file Image2.png]

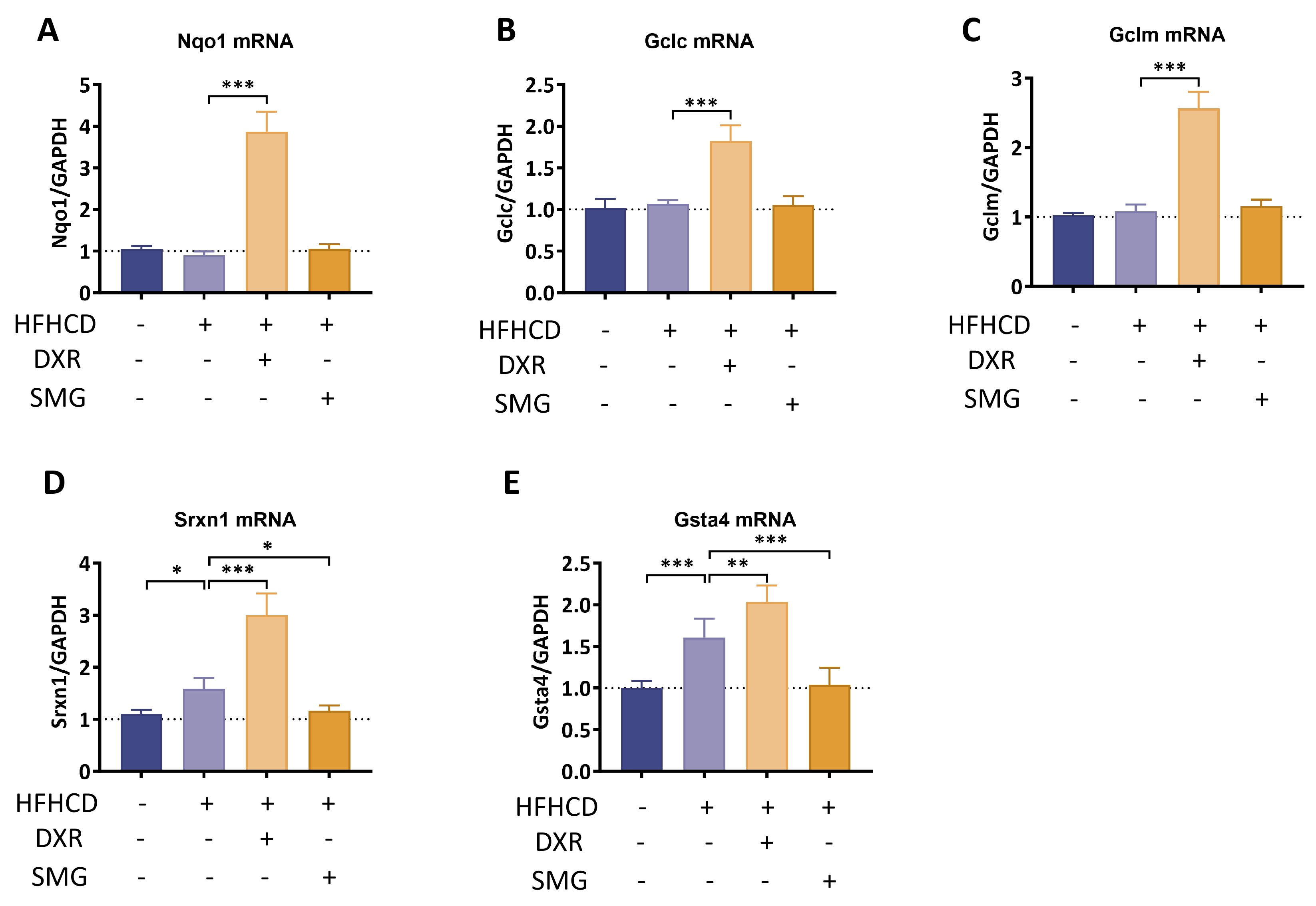

Supplement: Supplementary Figure 3 — DXR upregulates hepatic Nrf2 downstream antioxidant gene expression in HFHCD-fed mice. Relative mRNA expression levels of Nqo1 (A), Gclc (B), Gclm (C), Srxn1 (D), and Gsta4 (E) in mouse liver tissues were measured by qRT-PCR. Compared with the HFHCD group, DXR significantly increased the mRNA levels of Nqo1, Gclc, and Gclm. Srxn1 and Gsta4 were elevated in the HFHCD group and were further upregulated after DXR treatment. *P < 0.05; **P < 0.01; ***P < 0.001 vs. HFHCD. [file Image3.jpeg]

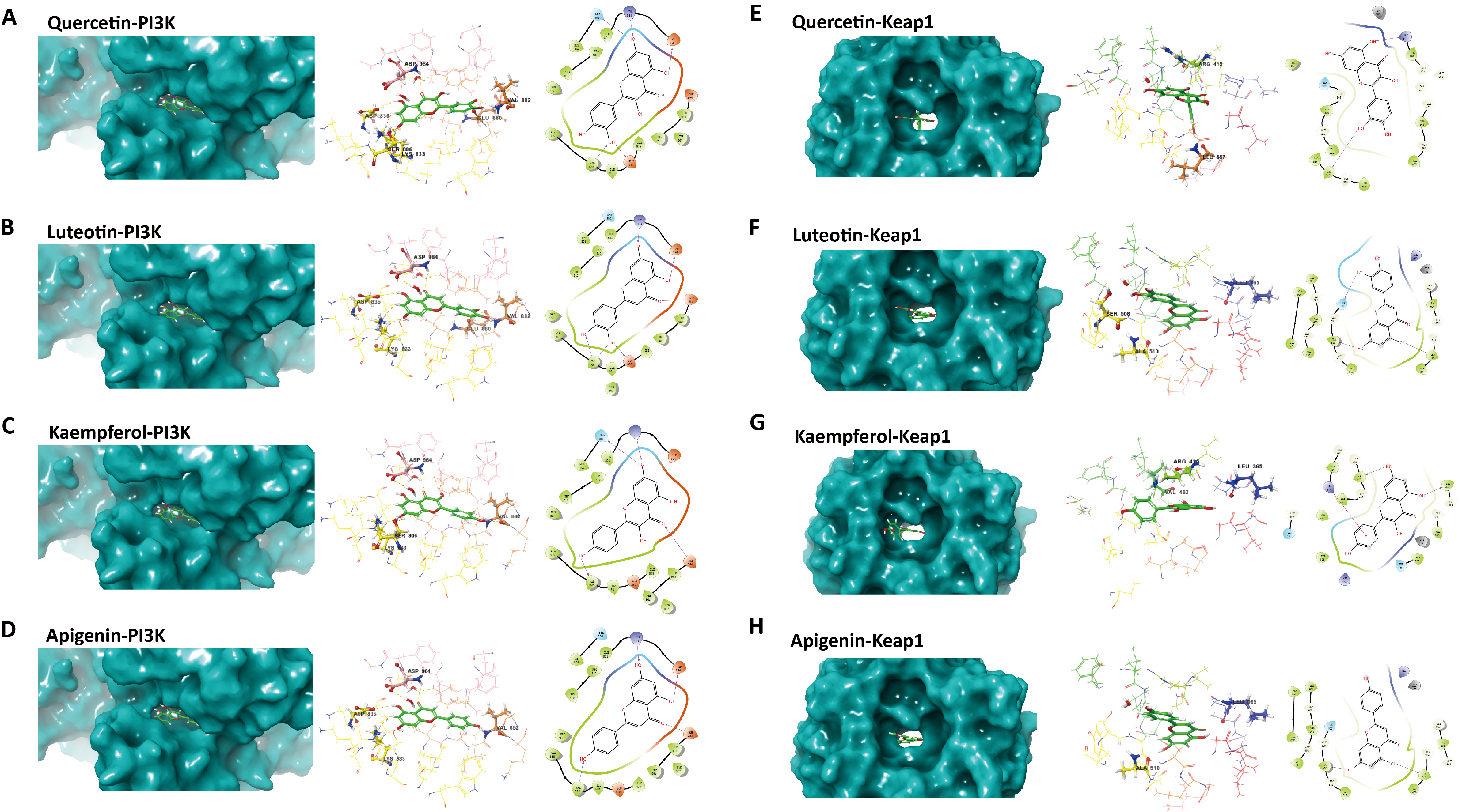

Supplement: Supplementary Figure 4 — The virtual molecular docking of PI3K and top 4 active components derived from DXR. The 3D docking illustration, 3D and 2D interaction diagrams of PI3K (PDB ID: 5JHA) with quercetin (A), luteolin (B), kaempferol (C), apigenin (D), respectively. The 3D docking illustration, 3D and 2D interaction diagrams of Keap1 (PDB ID: 8IVG) with quercetin (E), luteolin (F), kaempferol (G), apigenin (H), respectively. The yellow dotted line (middle) and purple arrow (right) represent the hydrogen bonds. [file Image4.png]
