# Supplementary material for: Daixie recipe ameliorates diet-induced MASH in mice via activating PI3K/AKT and Keap1/Nrf2 signaling
Source: Front Endocrinol (Lausanne). 2026 Mar 13;17:1772033. doi: 10.3389/fendo.2026.1772033 (PMC13021464; doi:10.3389/fendo.2026.1772033)
Supplement: Supplementary file 5 [file Table1.docx]

Research article

**Daixie** **recipe** **ameliorates diet-induced MASH in mice *via* activating PI3K/AKT and Keap1/Nrf2 signaling**

**Xiaoli He^1†^, Jiawen You^1†^, Yanyan Deng^2^, Yiren Hu^3^, Shenglan Qi^4^, Qian Li^2^, Yunyi Yang^1^, Xiaoxiao Qu^1^, Yanting Shao^1^, Xinyi Fu^1^, Shiyu Yang^1^, Zhiying Wang^1^, Yunhao Li^1^, Min Zheng^1^, Wei Liu^4^, Hongjie Yang^1^, Guangbo Ge^2*^, Zheng Yao^1*^, and Yanming He^1*^**

^1^Department of Endocrinology, Center of Experimental Animals, Yueyang Hospital of Integrated Traditional Chinese and Western Medicine, Shanghai University of Traditional Chinese Medicine, Shanghai, 200437, China;

^2^Shanghai Frontiers Science Center of TCM Chemical Biology; Institute of Interdisciplinary Integrative Medicine Research, Shanghai University of Traditional Chinese Medicine, Shanghai, 201203, China;

^3^Zhuanqiao Community Health Service Center, Shanghai, 201108, China;

^4^Key Laboratory of Liver and Kidney Diseases (Ministry of Education); Institute of Liver Diseases, Shuguang Hospital Affiliated to Shanghai University of Traditional Chinese Medicine, Shanghai, 201203, China

***Correspondence:**

E-mail: heyanming176@163.com (Yanming He); yaozheng8848@163.com (Zheng Yao); geguangbo@shutcm.edu.cn (Guangbo Ge);

^†^These authors have contributed equally to this work and share first authorship.

**Supplementary Table 1**

| Chinese name | Botanical name | English name | Parts used | Proportion(g) |
| --- | --- | --- | --- | --- |
| Huang qi | *Astragalus membranaceus* (Fisch.) Bunge | Astragali Radix | Root | 15 |
| Ling zhi | *Ganoderma lucidum* (Leyss. Ex Fr.) Karst. | Ganoderma Lucidum seu Japonicum | Sporocarp | 9 |
| Wu mei | *Prunus mume* Siebold & Zucc | Fructus Mume | Fruit | 9 |
| Nv zhen zi | *Ligustrum lucidum* Ait. | Fructus Ligustri lucidi | Fruit | 9 |
| Chuan xiong | *Ligusticum chuanxiong* Hort. | Chuanxiong Radix | Rhizome | 6 |
| Dang gui | *Angelica sinensis* (Oliv.) Diels | Angelicae Sinensis Radix | Root | 9 |
| Sang ye | *Morus alba* L. | Folium Mori | Leaf | 9 |
| Fu ling | *Poria cocos* (Schw.) Wolf. | Poria | Sclerotium | 15 |

**Table S1.** Composition of Daixie recipe (DXR).

**Supplementary Table 2**

|  | Antibodies | Anti-species | Inc. | Dilution | Lot. |
| --- | --- | --- | --- | --- | --- |
| Primary antibodies | p-AKT1 | Rabbit | Invitrogen | 1: 1000 | PA5-121271 |
|  | AKT1 | Mouse | Santa Cruz | 1: 1000 | sc-5298 |
|  | Nrf2 | Rabbit | Abcam | 1: 1000 | ab62352 |
|  | HO-1 | Rabbit | Abcam | 1: 1000 | ab68477 |
|  | GAPDH | Mouse | Proteintech | 1: 5000 | 60004-1-Ig |
|  | GAPDH | Rabbit | Proteintech | 1: 5000 | 10494-1-AP |
|  | β-actin | Mouse | Abcam | 1: 5000 | ab8226 |
| Secondary antibodies | Anti-mouse | Goat | Beyotime | 1: 1000 | A0216 |
|  | Anti-rabbit | Goat | Beyotime | 1: 1000 | A0208 |
|  | FITC-anti-rabbit | Goat | Abcam | 1: 3000 | ab6717 |

**Table S2.** The list of primary antibodies and secondary antibodies.

**Supplementary Table 3**

| Primers | Forward | Reverse |
| --- | --- | --- |
| *Acc1* | AACATCCCGCACCTTCTTCTAC | CTTCCACAAACCAGCGTCTC |
| *Cpt1a* | TCCACCGTTTGACTTGTGACCC | CCCTTTATCCATTAGGAGCCGAT |
| *Srebp1c* | AACCTCATCCGCCACCT | GGTAGACAACAGCCGCATC |
| *Scd1* | CAGTTCCTACACGACCACCACTA | GGACGGATGTCTTCTTCCAGAT |
| *Fasn* | ACCTCATCACTAGAAGCCACCAG | GTGGTACTTGGCCTTGGGTTTA |
| *Tnf-α* | AAAATTCGAGTGACAAGCCTGTAG | CCCTTGAAGAGAACCTGGGAGTAG |
| *Il-1β* | AATCTATACCTGTCCTGTGTAATGAAAGAC | TGGGTATTGCTTGGGATCCA |
| *Il-6* | CCTCTGGTCTCTGGAGTACC | GGAGAGCATTGGAAATTGGGG |
| *Nqo1* | AGGATGGGAGGTACTCGAATC | TGCTAGAGATGACTCGGAAGG |
| *Gclc* | CTACCACGCAGTCAAGGACC | CCTCCATTCAGTAACAACTGGAC |
| *Gclm* | AGGAGCTTCGGGACTGTATCC | GGAAACTCCCTGACTAAATCGG |
| *Srxn1* | CCCAGGGTGGCGACTACTA | GTGGACCTCACGAGCTTGG |
| *Gsta4* | TACCTCGCTGCCAAGTACAAC | GAGCCACGGCAATCATCATCA |
| *Gapdh* | CAACTTTGGCATTGTGGAAGG | ACACATTGGGGGTAGGAACAC |

**Table S3.** Real-time PCR primer sequences.

**Supplementary Table 4**

| **No.** | **RT/min** | **Ion model** | **Measured mass /Da** | **Calculated mass /Da** | **Error/ppm** | **Molecular formula** | **Component name** | **Source** | **Peak area** |
| --- | --- | --- | --- | --- | --- | --- | --- | --- | --- |
| 1 | 0.89 | [M-H]- | 191.0552 | 191.0550 | 1.180 | C7H12O6 | Hexahydro-1,3,4,5-Tetrahydroxybenzoic acid | Ligusticum chuanxiong Hort. (LC) | 1601504933 |
| 2 | 0.91 | [M+H]+ | 104.0710 | 104.0706 | 3.602 | C4H9NO2 | Gamma-aminobutyric acid | Morus alba L. (MA) | 73351582 |
| 3 | 0.92 | [M-H]- | 341.1091 | 341.1078 | 3.612 | C12H22O11 | Sucrose | Poria cocos (Schw.) Wolf. (PC) Angelica sinensis (Oliv.) Diels (AS) Prunus mume Siebold & Zucc. (PM) Morus alba L. (MA) | 2522117064 |
| 4 | 1.43 | [M+Na]+ | 159.0894 | 159.0893 | 1.008 | C8H12N2 | Tetramethylpyrazine | Ligusticum chuanxiong Hort. (LC) | 14604559 |
| 5 | 1.65 | [M-H]- | 117.0180 | 117.0182 | -2.266 | C4H6O4 | Succinic acid | Angelica sinensis (Oliv.) Diels (AS) | 334193451 |
| 6 | 2.03 | [M-H]- | 169.0133 | 169.0132 | 0.830 | C7H6O5 | Gallic acid | Ligusticum chuanxiong Hort. (LC) Prunus mume Siebold & Zucc. (PM) | 25517588 |
| 7 | 3.27 | [M-H]- | 101.0593 | 101.0597 | -3.622 | C5H10O2 | Valerenic acid | Prunus mume Siebold & Zucc. (PM) | 4726091 |
| 8 | 4.24 | [M-H]- | 153.0182 | 153.0182 | -0.491 | C7H6O4 | Protocatechuic acid | Ligusticum chuanxiong Hort. (LC) | 115179345 |
| 9 | 4.77 | [M-H]- | 255.0511 | 255.0499 | 4.591 | C11H12O7 | Piscidic acid | Prunus mume Siebold & Zucc. (PM) | 77885265 |
| 10 | 6.44 | [M+H]+ | 169.0495 | 169.0495 | -0.386 | C8H8O4 | Vanillic acid | Ligusticum chuanxiong Hort. (LC) | 113967924 |
| 11 | 6.46 | [M-H]- | 137.0231 | 137.0233 | -1.391 | C7H6O3 | 3,4-Dihydroxybenzaldehyde | Ligusticum chuanxiong Hort. (LC) | 229803884 |
| 12 | 6.46 | [M-H]- | 353.0880 | 353.0867 | 3.601 | C16H18O9 | Cryptochlorogenic acid | Morus alba L. (MA) | 1345133195 |
| 13 | 6.53 | [M-H]- | 153.0181 | 153.0182 | -0.181 | C7H6O4 | Gentisic acid | Prunus mume Siebold & Zucc. (PM) | 411585612 |
| 14 | 6.85 | [M-H]- | 181.0499 | 181.0495 | 2.070 | C9H10O4 | Dihydrocaffeic acid | Ligusticum chuanxiong Hort. (LC) | 15492762 |
| 15 | 7.44 | [M+H]+ | 421.1679 | 421.1646 | 8.014 | C25H24O6 | Morusin | Morus alba L. (MA) | 32006782 |
| 16 | 7.63 | [M-H]- | 451.1099 | 451.1082 | 3.743 | C17H24O14 | Nuezhenidic acid | Ligustrum lucidum Ait. (LL) | 43857015 |
| 17 | 8.03 | [M-H]- | 177.0185 | 177.0182 | 1.270 | C9H6O4 | 6,7-Dihydroxycoumarin | Astragalus membranaceus (Fisch.) Bunge (AM) | 10796653 |
| 18 | 8.62 | [M+Na]+ | 323.1097 | 323.1101 | -1.282 | C14H20O7 | Salidroside | Ligustrum lucidum Ait. (LL) | 4848108225 |
| 19 | 8.63 | [M-H]- | 367.1013 | 367.1024 | -2.911 | C17H20O9 | 5-O-Feruloylquinic acid | Astragalus membranaceus (Fisch.) Bunge (AM) | 7406767 |
| 20 | 9.04 | [M+H]+ | 304.1643 | 304.1645 | 1.021 | C15H10O7 | Quercetin | Morus alba L. (MA) Astragalus membranaceus (Fisch.) Bunge (AM) Ligustrum lucidum Ait. (LL) | 53329192 |
| 21 | 9.38 | [M-H]- | 177.0185 | 177.0182 | 1.383 | C9H6O4 | Esculetin | Prunus mume Siebold & Zucc. (PM) | 59622432 |
| 22 | 9.42 | [M+H]+ | 286.8437 | 286.8440 | 0.932 | C15H10O6 | Luteolin | Morus alba L. (MA) Astragalus membranaceus (Fisch.) Bunge (AM) Ligustrum lucidum Ait. (LL) | 25064727 |
| 23 | 9.60 | [M-H]- | 353.0881 | 353.0867 | 4.026 | C16H18O9 | Chlorogenic Acid | Morus alba L. (MA) Prunus mume Siebold & Zucc. (PM) | 871136423 |
| 24 | 9.65 | [M+H]+ | 195.0654 | 195.0652 | 0.998 | C10H10O4 | 3-Hydroxy-4-methoxycinnamic acid | Ligusticum chuanxiong Hort. (LC) | 26608938 |
| 25 | 9.67 | [M-H]- | 179.0340 | 179.0339 | 0.641 | C9H8O4 | Caffeic acid | Astragalus membranaceus (Fisch.) Bunge (AM) | 97807096 |
| 26 | 10.10 | [M-H]- | 367.1036 | 367.1024 | 3.245 | C17H20O9 | 4-O-feruloylquinic acid | Astragalus membranaceus (Fisch.) Bunge (AM) | 57405515 |
| 27 | 10.10 | [M-H]- | 567.1730 | 567.1708 | 3.770 | C26H32O14 | Mulberroside A | Morus alba L. (MA) | 1559328 |
| 28 | 10.15 | [M+H]+ | 625.1762 | 625.1763 | -0.162 | C28H32O16 | Complanatoside A | Astragalus membranaceus (Fisch.) Bunge (AM) | 17506966 |
| 29 | 10.53 | [M-H]- | 353.0879 | 353.0867 | 3.431 | C16H18O9 | Isochlorogenic acid | Morus alba L. (MA) Ligustrum lucidum Ait. (LL) | 1110978370 |
| 30 | 10.81 | [M-H]- | 477.1617 | 477.1603 | 2.898 | C20H30O13 | Kelampayoside A | Morus alba L. (MA) | 36588952 |
| 31 | 11.03 | [M-H]- | 315.1090 | 315.1074 | 4.875 | C14H20O8 | Cimidahurinine | Ligustrum lucidum Ait. (LL) | 195106770 |
| 32 | 11.26 | [M+H]+ | 407.1313 | 407.1337 | -5.892 | C20H22O9 | trans-Resveratrol 4'-O-glucuronide | Morus alba L. (MA) | 19358640 |
| 33 | 11.41 | [M+H]+ | 153.0546 | 153.0546 | -0.331 | C8H8O3 | Vanillin | Prunus mume Siebold & Zucc. (PM) Ganoderma lucidum (Leyss. Ex Fr.) Karst. (GL) | 209637408 |
| 34 | 11.46 | [M-H]- | 367.1038 | 367.1024 | 3.818 | C17H20O9 | 4-O-Feruloylquinic acid | Ligusticum chuanxiong Hort. (LC) | 8841004 |
| 35 | 21.85 | [M+H]+ | 286.8695 | 286.8693 | -0.239 | C15H10O6 | Kaempferol | Morus alba L. (MA) Astragalus membranaceus (Fisch.) Bunge (AM) Ligustrum lucidum Ait. (LL) | 2694939 |
| 36 | 12.14 | [M-H]- | 625.1417 | 625.1399 | 2.870 | C27H30O17 | Quercetin-O-sucroside | Morus alba L. (MA) | 27735786 |
| 37 | 12.20 | [M+H]+ | 271.0600 | 271.0601 | -0.258 | C15H10O5 | Apigenin | Morus alba L. (MA) Astragalus membranaceus (Fisch.) Bunge (AM) Ligustrum lucidum Ait. (LL) | 2807224 |
| 38 | 12.58 | [M-H]- | 567.1727 | 567.1708 | 3.347 | C26H32O14 | cis-Mulberroside A | Morus alba L. (MA) | 1932603 |
| 39 | 12.79 | [M-H]- | 447.1517 | 447.1497 | 4.400 | C19H28O12 | 3,4-Dimethoxyphenyl beta-D-glucoside | Morus alba L. (MA) | 1623747433 |
| 40 | 13.13 | [M+H]+ | 163.0753 | 163.0754 | -0.222 | C10H10O2 | Isosafrole | Ligusticum chuanxiong Hort. (LC) | 125728484 |
| 41 | 13.51 | [M-H]- | 565.1570 | 565.1552 | 3.182 | C26H30O14 | Mulberroside F | Morus alba L. (MA) | 2420882 |
| 42 | 13.55 | [M-H]- | 583.2045 | 583.2021 | 4.009 | C27H36O14 | Lucidumoside C | Ligustrum lucidum Ait. (LL) | 7037680 |
| 43 | 13.73 | [M-H]- | 367.1037 | 367.1024 | 3.572 | C17H20O9 | 3-O-Feruloylquinic acid | Ligusticum chuanxiong Hort. (LC) | 35696668 |
| 44 | 13.80 | [M-H]- | 551.1775 | 551.1759 | 2.853 | C26H32O13 | Mulberroside E | Morus alba L. (MA) | 13498279 |
| 45 | 13.98 | [M-H]- | 609.1466 | 609.1450 | 2.625 | C27H30O16 | Kaempferol 3-O-sophoroside | Ligustrum lucidum Ait. (LL) | 18031805 |
| 46 | 14.05 | [M-H]- | 367.1036 | 367.1024 | 3.491 | C17H20O9 | 5-O-Feruloylquinic acid | Ligusticum chuanxiong Hort. (LC) | 49499942 |
| 47 | 14.14 | [M+H]+ | 255.0628 | 255.0652 | -9.313 | C15H10O4 | Daidzein | Ligustrum lucidum Ait. (LL) | 5121733 |
| 48 | 14.68 | [M+H]+ | 301.0704 | 301.0707 | -0.879 | C16H12O6 | Rhamnocitrin | Morus alba L. (MA) | 25482613 |
| 49 | 14.68 | [M+H]+ | 463.1230 | 463.1235 | -1.010 | C22H22O11 | Kaempferide | Astragalus membranaceus (Fisch.) Bunge (AM) | 12883680 |
| 50 | 14.89 | [M+H]+ | 195.0652 | 195.0652 | 0.075 | C10H10O4 | Ferulic acid | Ligusticum chuanxiong Hort. (LC) | 181077197 |
| 51 | 15.18 | [M+H]+ | 229.0859 | 229.0859 | -0.265 | C14H12O3 | Resveratrol | Ligustrum lucidum Ait. (LL) Morus alba L. (MA) | 60396564 |
| 52 | 16.53 | [M-H]- | 729.2260 | 729.2237 | 3.147 | C32H42O19 | 3-O-β-D-Glucopyranosyl-2'-hydroxyverbascoside 4'-(6-O-β-D-glucopyranosyl-β-D-glucopyranoside) | Morus alba L. (MA) | 1990120 |
| 53 | 16.55 | [M+H]+ | 515.1521 | 515.1548 | -5.218 | C26H26O11 | Calycosin-7-O-β-D-｛6"[(E)-but-2-enoy I]｝-glucoside | Astragalus membranaceus (Fisch.) Bunge (AM) | 7382647 |
| 54 | 16.69 | [M+H]+ | 257.0808 | 257.0808 | -0.332 | C15H12O4 | (Z)-1-(2,4-dihydroxyphenyl)-3-(4-hydroxyphenyl)prop-2-en-1-one | Morus alba L. (MA) | 12260387 |
| 55 | 16.96 | [M-H]- | 729.2259 | 729.2237 | 3.065 | C32H42O19 | 4'-O-β-D-Glucopyranosyl-2'-hydroxyverbascoside 3-(6-O-β-D-glucopyranosyl-β-D-glucopyranoside) | Morus alba L. (MA) | 8609937 |
| 56 | 17.11 | [M+HCOOH]- | 491.1197 | 491.1184 | 2.560 | C22H22O10 | Eriocricoside glucoside | Astragalus membranaceus (Fisch.) Bunge (AM) | 1081831899 |
| 57 | 17.12 | [M+H]+ | 285.0752 | 285.0758 | -1.824 | C16H12O5 | Calycosin | Astragalus membranaceus (Fisch.) Bunge (AM) | 28804944 |
| 58 | 17.14 | [M-H]- | 341.1006 | 341.1020 | -4.030 | C19H18O6 | Tetramethoxyluteolin | Ganoderma lucidum (Leyss. Ex Fr.) Karst. (GL) | 27833476 |
| 59 | 17.23 | [M+H]+ | 227.1276 | 227.1278 | -0.773 | C12H18O4 | Senkyunolide J | Ligusticum chuanxiong Hort. (LC) | 187145226 |
| 60 | 17.24 | [M+HCOOH]- | 477.1041 | 477.1028 | 2.741 | C21H20O10 | Genistin | Astragalus membranaceus (Fisch.) Bunge (AM) | 4651110 |
| 61 | 17.32 | [M+H]+ | 209.1171 | 209.1172 | -0.578 | C12H16O3 | Senkyunolide G | Ligusticum chuanxiong Hort. (LC) | 434398157 |
| 62 | 17.60 | [M-H]- | 329.0679 | 329.0656 | 7.144 | C17H14O7 | Iristectorigenin A | Ganoderma lucidum (Leyss. Ex Fr.) Karst. (GL) | 3554555 |
| 63 | 17.99 | [M-H]- | 609.18311 | 609.1814 | 2.813 | C28H34O15 | Hesperidin | Ligustrum lucidum Ait. (LL) | 13357313 |
| 64 | 18.25 | [M-H]- | 239.0924 | 239.0914 | 3.973 | C12H16O5 | Senkyunolide S | Ligusticum chuanxiong Hort. (LC) | 44476152 |
| 65 | 18.50 | [M-H]- | 609.1832 | 609.1814 | 2.911 | C28H34O15 | Neohesperidin | Ligustrum lucidum Ait. (LL) | 22482978 |
| 66 | 18.77 | [M+H]+ | 225.1121 | 225.1121 | -0.335 | C12H16O4 | Senkyunolide I | Ligusticum chuanxiong Hort. (LC) | 14468575 |
| 67 | 18.79 | [M-H]- | 555.1722 | 555.1708 | 2.428 | C25H32O14 | Ligustaloside A | Ligustrum lucidum Ait. (LL) | 24702110 |
| 68 | 18.82 | [M-H]- | 701.2308 | 701.2287 | 2.894 | C31H42O18 | Neonuezhenide | Ligustrum lucidum Ait. (LL) | 59717962 |
| 69 | 18.84 | [M+H]+ | 725.2258 | 725.2287 | -4.096 | C33H40O18 | Ligustroflavone | Ligustrum lucidum Ait. (LL) | 89526082 |
| 70 | 18.90 | [M-H]- | 609.1466 | 609.1450 | 2.625 | C27H30O16 | Rutin | Morus alba L. (MA) Ligustrum lucidum Ait. (LL) | 8665257 |
| 71 | 19.11 | [M-H]- | 623.1987 | 623.1970 | 2.605 | C29H36O15 | Verbascoside | Ligustrum lucidum Ait. (LL) | 45438674 |
| 72 | 19.22 | [M-H]- | 239.0899 | 239.0914 | -6.190 | C12H16O5 | Senkyunolide R | Ligusticum chuanxiong Hort. (LC) | 33224956 |
| 73 | 19.56 | [M+H]+ | 463.1232 | 463.1235 | -0.557 | C22H22O11 | Pratensein 7-O-glucopyranoside | Astragalus membranaceus (Fisch.) Bunge (AM) | 36184623 |
| 74 | 19.69 | [M-H]- | 431.0986 | 431.0973 | 3.147 | C21H20O10 | Vitexin | Prunus mume Siebold & Zucc. (PM) | 1807671 |
| 75 | 19.94 | [M-H]- | 553.1568 | 553.1552 | 2.925 | C25H30O14 | Ligustrosidic acid | Ligustrum lucidum Ait. (LL) | 28846257 |
| 76 | 20.10 | [M+H]+ | 207.1015 | 207.1016 | -0.535 | C12H14O3 | 4-Hydroxy-3-Butylphthalide | Ligusticum chuanxiong Hort. (LC) | 4977912813 |
| 77 | 20.10 | [M+H]+ | 225.1120 | 225.1121 | -0.558 | C12H16O4 | Senkyunolide H | Ligusticum chuanxiong Hort. (LC) | 78933245 |
| 78 | 20.56 | [M+Na]+ | 352.1540 | 352.1519 | 5.766 | C19H23NO4 | Isosinomenine | Ligustrum lucidum Ait. (LL) | 49036923 |
| 79 | 20.61 | [M+HCOOH]- | 695.3972 | 695.4001 | -4.233 | C36H58O10 | Huangqiyenins A | Astragalus membranaceus (Fisch.) Bunge (AM) | 12168220 |
| 80 | 20.71 | [M-H]- | 685.2352 | 685.2338 | 2.049 | C31H42O17 | Specneuzhenide | Ligustrum lucidum Ait. (LL) | 3541240444 |
| 81 | 21.48 | [M+H]+ | 533.1291 | 533.1290 | 0.230 | C25H24O13 | Calycosin-7-O-β-D-glucoside | Astragalus membranaceus (Fisch.) Bunge (AM) | 13690761 |
| 82 | 21.66 | [M+H]+ | 255.0647 | 255.0652 | -1.785 | C15H10O4 | 7,4'-Dihydroxyflavone | Astragalus membranaceus (Fisch.) Bunge (AM) | 1158721 |
| 83 | 22.25 | [M+HCOOH]- | 475.1248 | 475.1235 | 2.741 | C22H22O9 | Ononin | Astragalus membranaceus (Fisch.) Bunge (AM) | 570325382 |
| 84 | 22.27 | [M+H]+ | 431.1335 | 431.1337 | -0.275 | C22H22O9 | Ononin | Astragalus membranaceus (Fisch.) Bunge (AM) | 738633961 |
| 85 | 22.34 | [M+H]+ | 139.1118 | 139.1117 | 0.132 | C9H14O | Ligustral | Ligustrum lucidum Ait. (LL) | 56211598 |
| 86 | 22.54 | [M-H]- | 539.1774 | 539.1759 | 2.806 | C25H32O13 | Oleuropein | Ligustrum lucidum Ait. (LL) | 34649802 |
| 87 | 22.85 | [M+H]+ | 301.1067 | 301.1071 | -1.096 | C17H16O5 | Medicarpin | Astragalus membranaceus (Fisch.) Bunge (AM) | 1315156793 |
| 88 | 22.86 | [M-H]- | 147.0803 | 147.0804 | -1.098 | C10H12O | Anethole | Angelica sinensis (Oliv.) Diels (AS) | 46078427 |
| 89 | 22.86 | [M-H]- | 685.2353 | 685.2338 | 2.136 | C31H42O17 | Excelsioside O-beta-D-glucopyranoside | Ligustrum lucidum Ait. (LL) | 530071807 |
| 90 | 22.90 | [M-H]- | 287.0538 | 287.0550 | -4.321 | C15H12O6 | Eriodictyol | Astragalus membranaceus (Fisch.) Bunge (AM) | 17754384 |
| 91 | 23.27 | [M+H]+ | 489.1388 | 489.1391 | -0.711 | C24H24O11 | Calycosin-7-O-β-D-(6"-acetyl)-glucoside | Astragalus membranaceus (Fisch.) Bunge (AM) | 99557381 |
| 92 | 23.49 | [M+Na]+ | 547.1785 | 547.1786 | -0.123 | C25H32O12 | Ligustroside | Ligustrum lucidum Ait. (LL) | 416614065 |
| 93 | 24.26 | [M-H]- | 685.2357 | 685.2338 | 2.764 | C31H42O17 | Nuezhenide | Ligustrum lucidum Ait. (LL) | 205142065 |
| 94 | 24.33 | [M-H]- | 203.0708 | 203.0703 | 2.409 | C12H12O3 | Senkyunolide B | Ligusticum chuanxiong Hort. (LC) | 23803655 |
| 95 | 24.42 | [M+H]+ | 423.1774 | 423.1802 | -6.581 | C25H26O6 | Mulberrin | Morus alba L. (MA) | 4279944 |
| 96 | 24.60 | [M+H]+ | 285.0755 | 285.0758 | -0.772 | C16H12O5 | Biochanin A | Astragalus membranaceus (Fisch.) Bunge (AM) | 55549556 |
| 97 | 25.00 | [M+H]+ | 303.1225 | 303.1227 | -0.561 | C17H18O5 | Mucronulatol | Astragalus membranaceus (Fisch.) Bunge (AM) | 206579376 |
| 98 | 25.02 | [M-H]- | 463.1613 | 463.1599 | 3.059 | C23H28O10 | Isomucronulatol 7-O-glucoside | Astragalus membranaceus (Fisch.) Bunge (AM) | 339557835 |
| 99 | 25.66 | [M-H]- | 221.0816 | 221.0808 | 3.323 | C12H14O4 | 3-butyl-4,7-dihydroxy-3H-2-benzofuran-1-one | Ligusticum chuanxiong Hort. (LC) | 27231363 |
| 100 | 25.93 | [M+HCOOH]- | 315.0878 | 315.0863 | 4.809 | C16H14O4 | 2'-O-Methylisoliquiritigenin | Astragalus membranaceus (Fisch.) Bunge (AM) | 5247015 |
| 101 | 26.86 | [M-H]- | 221.0815 | 221.0808 | 2.916 | C12H14O4 | Ethyl ferulate | Ligusticum chuanxiong Hort. (LC) | 8240896 |
| 102 | 27.36 | [M-H]- | 271.0613 | 271.0601 | 4.464 | C15H12O5 | Genistein | Astragalus membranaceus (Fisch.) Bunge (AM) | 1306852 |
| 103 | 27.45 | [M+Na]+ | 1095.3521 | 1095.3527 | -0.609 | C48H64O27 | Nuezhenoside G13 | Ligustrum lucidum Ait. (LL) | 1242986142 |
| 104 | 27.49 | [M+Na]+ | 607.2018 | 607.1997 | 3.480 | C27H36O14 | Lucidumoside C | Ligustrum lucidum Ait. (LL) | 6293375 |
| 105 | 27.54 | [M-H]- | 529.2809 | 529.2796 | 2.542 | C30H42O8 | Ganoderic acid D2 | Ganoderma lucidum (Leyss. Ex Fr.) Karst. (GL) | 34114613 |
| 106 | 27.56 | [M+H]+ | 189.0909 | 189.0910 | -0.826 | C12H12O2 | (Z)-Butylidenephthalide | Ligusticum chuanxiong Hort. (LC) | 539722094 |
| 107 | 27.60 | [M+Na]+ | 527.1519 | 527.1524 | -0.878 | C25H28O11 | (-)-Methylnissolin-3-O-β-D-(6'-acetyl)-glucoside | Astragalus membranaceus (Fisch.) Bunge (AM) | 144842608 |
| 108 | 27.88 | [M+H]+ | 301.1068 | 301.1071 | -0.997 | C17H16O5 | (6αR,11αR) 3,9-dimethoxy-10-hydroxypterocaroan | Astragalus membranaceus (Fisch.) Bunge (AM) | 101755862 |
| 109 | 27.95 | [M+H]+ | 191.10651 | 191.10665 | -0.765 | C12H14O2 | dl-3n-butylphthalide | Ligusticum chuanxiong Hort. (LC) | 587419570 |
| 110 | 28.04 | [M+HCOOH]- | 532.3000 | 532.2957 | 5.248 | C30H48O3 | Oleanolic acid | Ligustrum lucidum Ait. (LL) Astragalus membranaceus (Fisch.) Bunge (AM) Ganoderma lucidum (Leyss. Ex Fr.) Karst. (GL) | 13521052 |
| 111 | 28.19 | [M-H]- | 529.2808 | 529.2796 | 2.202 | C30H42O8 | Ganoderic acid C6 | Ganoderma lucidum (Leyss. Ex Fr.) Karst. (GL) | 280893312 |
| 112 | 28.79 | [M+Na]+ | 1095.3522 | 1095.3527 | -0.500 | C48H64O27 | Oleonuezhenide | Ligustrum lucidum Ait. (LL) | 200581666 |
| 113 | 29.22 | [M+H]+ | 341.1357 | 341.1384 | -7.827 | C20H20O5 | Euchrenone A | Morus alba L. (MA) | 19704144 |
| 114 | 29.31 | [M-H]- | 531.2966 | 531.2952 | 2.494 | C30H44O8 | Ganoderic acid G | Ganoderma lucidum (Leyss. Ex Fr.) Karst. (GL) | 197800270 |
| 115 | 29.31 | [M+HCOOH]- | 532.3000 | 532.2957 | 5.248 | C30H48O3 | Ursolic acid | Morus alba L. (MA) Ligustrum lucidum Ait. (LL) Ganoderma lucidum (Leyss. Ex Fr.) Karst. (GL) | 66225200 |
| 116 | 29.35 | [M-H]- | 513.2860 | 513.2847 | 2.591 | C30H42O7 | Ganoderenic acid A | Ganoderma lucidum (Leyss. Ex Fr.) Karst. (GL) | 822525855 |
| 117 | 29.68 | [M+H]+ | 303.1223 | 303.1227 | -1.188 | C17H18O5 | Isomucronulatol | Astragalus membranaceus (Fisch.) Bunge (AM) | 13509294 |
| 118 | 29.70 | [M-H]- | 267.0664 | 267.0652 | 4.473 | C16H12O4 | Formononetin | Astragalus membranaceus (Fisch.) Bunge (AM) | 57817780 |
| 119 | 29.70 | [M-H]- | 513.2860 | 513.2847 | 2.591 | C30H42O7 | Ganoderic acid LM2 | Ganoderma lucidum (Leyss. Ex Fr.) Karst. (GL) | 219448826 |
| 120 | 29.72 | [M+H]+ | 269.0805 | 269.0808 | -1.358 | C16H12O4 | Formononetin | Astragalus membranaceus (Fisch.) Bunge (AM) | 33774966 |
| 121 | 29.77 | [M-H]- | 515.3016 | 515.3003 | 2.406 | C30H44O7 | Ganoderic acid B | Ganoderma lucidum (Leyss. Ex Fr.) Karst. (GL) | 282623323 |
| 122 | 29.90 | [M+H]+ | 193.1223 | 193.1223 | -0.188 | C12H16O2 | Senkyunolide A | Ligusticum chuanxiong Hort. (LC) | 1130276439 |
| 123 | 30.16 | [M-H]- | 573.3070 | 573.3058 | 1.990 | C32H46O9 | Ganoderic acid K | Ganoderma lucidum (Leyss. Ex Fr.) Karst. (GL) | 173083020 |
| 124 | 30.63 | [M-H]- | 529.2809 | 529.2796 | 2.542 | C30H42O8 | Ganoderic acid N | Ganoderma lucidum (Leyss. Ex Fr.) Karst. (GL) | 123537859 |
| 125 | 30.69 | [M+Na]+ | 595.2875 | 595.2878 | -0.460 | C32H44O9 | Ganoderic acid H | Ganoderma lucidum (Leyss. Ex Fr.) Karst. (GL) | 1286003975 |
| 126 | 30.74 | [M-H]- | 531.2968 | 531.2952 | 2.833 | C30H44O8 | Ganoderic acid I | Ganoderma lucidum (Leyss. Ex Fr.) Karst. (GL) | 12938482 |
| 127 | 30.99 | [M-H]- | 203.0708 | 203.0703 | 2.075 | C12H12O3 | Senkyunolide E | Ligusticum chuanxiong Hort. (LC) | 3632186 |
| 128 | 30.99 | [M-H]- | 513.2861 | 513.2847 | 2.708 | C30H42O7 | Ganoderic acid C1 | Ganoderma lucidum (Leyss. Ex Fr.) Karst. (GL) | 209833390 |
| 129 | 31.08 | [M+H]+ | 317.1357 | 317.1384 | -8.231 | C18H20O5 | 7-O-methylisomucronulatol | Astragalus membranaceus (Fisch.) Bunge (AM) | 56026439 |
| 130 | 31.37 | [M+Na]+ | 553.1676 | 553.1680 | -0.782 | C27H30O11 | (-)-Methylnissolin 3-O-β-D-｛6'-[(E)-but-2-enoyl]｝-glucoside | Astragalus membranaceus (Fisch.) Bunge (AM) | 4842636 |
| 131 | 31.60 | [M-H]- | 513.2858 | 513.2847 | 2.124 | C30H42O7 | Ganoderic acid D | Ganoderma lucidum (Leyss. Ex Fr.) Karst. (GL) | 259942729 |
| 132 | 31.78 | [M-H]- | 515.3015 | 515.3003 | 2.173 | C30H44O7 | Ganoderic acid A | Ganoderma lucidum (Leyss. Ex Fr.) Karst. (GL) | 993666584 |
| 133 | 31.99 | [M-H]- | 569.2759 | 569.2745 | 2.408 | C32H42O9 | Ganoderic acid F | Ganoderma lucidum (Leyss. Ex Fr.) Karst. (GL) | 431842412 |
| 134 | 32.14 | [M-H]- | 513.2860 | 513.2847 | 2.474 | C30H42O7 | Ganoderic acid Z | Ganoderma lucidum (Leyss. Ex Fr.) Karst. (GL) | 23004152 |
| 135 | 32.19 | [M+H]+ | 191.1065 | 191.1067 | -0.713 | C12H14O2 | 3-Butylphthalide | Ligusticum chuanxiong Hort. (LC) | 87049312 |
| 136 | 32.79 | [M+HCOOH]- | 502.3260 | 502.3216 | 5.977 | C30H50O | Lupeol | Angelica sinensis (Oliv.) Diels (AS) Astragalus membranaceus (Fisch.) Bunge (AM) Ganoderma lucidum (Leyss. Ex Fr.) Karst. (GL) | 2995957 |
| 137 | 33.02 | [M+H]+ | 279.1588 | 279.1591 | -0.880 | C16H22O4 | Dibutyl phthalate | Ligusticum chuanxiong Hort. (LC) | 82235974 |
| 138 | 33.17 | [M+H]+ | 191.1065 | 191.1067 | -0.713 | C12H14O2 | (Z)-Ligustilide | Ligusticum chuanxiong Hort. (LC) | 112335908 |
| 139 | 34.12 | [M-H]- | 513.2857 | 513.2847 | 2.007 | C30H42O7 | Ganoderic acid J | Ganoderma lucidum (Leyss. Ex Fr.) Karst. (GL) | 134878884 |
| 140 | 34.39 | [M+H]+ | 311.1250 | 311.1278 | -8.857 | C19H18O4 | Moracin C | Morus alba L. (MA) | 8402565 |
| 141 | 35.33 | [M+Na]+ | 577.3518 | 577.3500 | 3.100 | C34H50O6 | Ganodermic acid S | Ganoderma lucidum (Leyss. Ex Fr.) Karst. (GL) | 987688 |
| 142 | 36.73 | [M+Na]+ | 809.4650 | 809.4658 | -0.924 | C41H70O14 | Astramembranosides B | Astragalus membranaceus (Fisch.) Bunge (AM) | 27689014 |
| 143 | 36.89 | [M+HCOOH]- | 991.5126 | 991.5108 | 1.810 | C47H78O19 | Astragaloside VII | Astragalus membranaceus (Fisch.) Bunge (AM) | 2491585 |
| 144 | 37.05 | [M+Na]+ | 645.3970 | 645.3973 | -0.425 | C35H58O9 | Astramembrannin II | Astragalus membranaceus (Fisch.) Bunge (AM) | 21158663 |
| 145 | 37.34 | [M+Na]+ | 511.3393 | 511.3394 | -0.207 | C30H48O5 | Astragaloside I | Astragalus membranaceus (Fisch.) Bunge (AM) | 39008414 |
| 146 | 37.42 | [M+Na]+ | 675.4076 | 675.4079 | -0.413 | C36H60O10 | Astraverrucin I | Astragalus membranaceus (Fisch.) Bunge (AM) | 104295448 |
| 147 | 37.45 | [M+Na]+ | 809.4650 | 809.4658 | -0.998 | C41H70O14 | Cyclocanthosides E | Astragalus membranaceus (Fisch.) Bunge (AM) | 89633318 |
| 148 | 37.51 | [M-H]- | 669.4229 | 669.4208 | 3.094 | C36H62O11 | Mongholicoside B | Astragalus membranaceus (Fisch.) Bunge (AM) | 2009663 |
| 149 | 37.74 | [M+Na]+ | 809.4651 | 809.4658 | -0.850 | C41H70O14 | Isocyclocanthosides E | Astragalus membranaceus (Fisch.) Bunge (AM) | 29390304 |
| 150 | 38.09 | [M-H]- | 669.4229 | 669.4208 | 3.004 | C36H62O11 | Mongholicoside A | Astragalus membranaceus (Fisch.) Bunge (AM) | 7431734 |
| 151 | 39.71 | [M+HCOOH]- | 991.5125 | 991.5108 | 1.689 | C47H78O19 | Astragaloside V | Astragalus membranaceus (Fisch.) Bunge (AM) | 10914028 |
| 152 | 39.91 | [M+Na]+ | 933.4812 | 933.4818 | -0.666 | C47H74O17 | Acetylastragaloside I | Astragalus membranaceus (Fisch.) Bunge (AM) | 793091 |
| 153 | 39.93 | [M+HCOOH]- | 913.4817 | 913.4791 | 2.790 | C45H72O16 | Neoastragaloside I | Astragalus membranaceus (Fisch.) Bunge (AM) | 3702707 |
| 154 | 40.02 | [M+Na]+ | 513.3544 | 513.3551 | -1.200 | C30H50O5 | Isoastragaloside VII | Astragalus membranaceus (Fisch.) Bunge (AM) | 6049125 |
| 155 | 40.04 | [M+HCOOH]- | 829.4602 | 829.4580 | 2.650 | C41H68O14 | Isoastragaloside IV | Astragalus membranaceus (Fisch.) Bunge (AM) | 234395295 |
| 156 | 40.19 | [M+HCOOH]- | 943.5183 | 943.5261 | -8.311 | C47H78O16 | Isoastragaloside VI or Astragaloside VI | Astragalus membranaceus (Fisch.) Bunge (AM) | 18006065 |
| 157 | 40.29 | [M-H]- | 911.5010 | 911.4999 | 1.210 | C47H76O17 | Astragaloside VIII | Astragalus membranaceus (Fisch.) Bunge (AM) | 10775803 |
| 158 | 40.33 | [M+HCOOH]- | 829.4599 | 829.4580 | 2.276 | C41H68O14 | Astragaloside IV | Astragalus membranaceus (Fisch.) Bunge (AM) | 601937769 |
| 159 | 40.44 | [M+HCOOH]- | 1075.5337 | 1075.5320 | 1.604 | C51H82O21 | Agroastragaloside III | Astragalus membranaceus (Fisch.) Bunge (AM) | 4394600 |
| 160 | 40.61 | [M+HCOOH]- | 871.4703 | 871.4686 | 2.023 | C43H70O15 | Astragaloside II | Astragalus membranaceus (Fisch.) Bunge (AM) | 707243919 |
| 161 | 40.63 | [M+Na]+ | 645.3969 | 645.3973 | -0.704 | C35H58O9 | Atramembrannin | Astragalus membranaceus (Fisch.) Bunge (AM) | 93708713 |
| 162 | 40.84 | [M+Na]+ | 513.3549 | 513.3551 | -0.245 | C30H50O5 | Cycloastragenol | Astragalus membranaceus (Fisch.) Bunge (AM) | 7436863 |
| 163 | 40.89 | [M+HCOOH]- | 871.4705 | 871.4686 | 2.230 | C43H70O15 | Isoastragaloside II | Astragalus membranaceus (Fisch.) Bunge (AM) | 180330659 |
| 164 | 41.07 | [M+HCOOH]- | 913.4810 | 913.4791 | 2.056 | C45H72O16 | Astragaloside I | Astragalus membranaceus (Fisch.) Bunge (AM) | 83500647 |
| 165 | 41.08 | [M+Na]+ | 963.4907 | 963.4924 | -1.792 | C48H76O18 | Astraisoolesaponins A | Astragalus membranaceus (Fisch.) Bunge (AM) | 6453855 |
| 166 | 41.13 | [M-H]- | 379.1917 | 379.1904 | 3.492 | C24H28O4 | Levistolide A | Ligusticum chuanxiong Hort. (LC) | 4500029 |
| 167 | 41.20 | [M-H]- | 355.1552 | 355.1540 | 3.237 | C21H24O5 | Myricanone | Poria cocos (Schw.) Wolf. (PC) Ganoderma lucidum (Leyss. Ex Fr.) Karst. (GL) | 3688192 |
| 168 | 41.22 | [M+H]+ | 279.2316 | 279.2319 | -0.919 | C18H30O2 | linolenic acid | Ganoderma lucidum (Leyss. Ex Fr.) Karst. (GL) Ligustrum lucidum Ait. (LL) | 106173008 |
| 169 | 41.27 | [M+HCOOH]- | 913.4813 | 913.4791 | 2.385 | C45H72O16 | Isoastragaloside II | Astragalus membranaceus (Fisch.) Bunge (AM) | 6085343 |
| 170 | 41.28 | [M-H]- | 199.1697 | 199.1693 | 2.026 | C12H24O2 | Lauric acid | Ganoderma lucidum (Leyss. Ex Fr.) Karst. (GL) Prunus mume Siebold & Zucc. (PM) | 9796208 |
| 171 | 41.32 | [M+H]+ | 205.1227 | 205.1951 | 1.676 | C15H24 | α-humulene | Ligusticum chuanxiong Hort. (LC) Ganoderma lucidum (Leyss. Ex Fr.) Karst. (GL) | 4598993 |
| 172 | 41.34 | [M-H]- | 379.1892 | 379.1904 | -3.259 | C24H28O4 | Angelicide | Ligusticum chuanxiong Hort. (LC) | 24001128 |
| 173 | 41.39 | [M+Na]+ | 293.2446 | 293.2451 | -1.608 | C17H34O2 | Methyl palmitate | Poria cocos (Schw.) Wolf. (PC) Ligustrum lucidum Ait. (LL) Ganoderma lucidum (Leyss. Ex Fr.) Karst. (GL) | 27002982 |
| 174 | 41.83 | [M-H]- | 303.2330 | 303.2319 | 3.869 | C20H32O2 | Arachidonic acid | Ganoderma lucidum (Leyss. Ex Fr.) Karst. (GL) Prunus mume Siebold & Zucc. (PM) | 13202319 |
| 175 | 42.90 | [M-H]- | 281.2485 | 281.2475 | 3.674 | C18H34O2 | Oleic acid | Poria cocos (Schw.) Wolf. (PC) Ligustrum lucidum Ait. (LL) Ganoderma lucidum (Leyss. Ex Fr.) Karst. (GL) Prunus mume Siebold & Zucc. (PM) | 40441305 |

**Table S4.** Compounds identified in DXR.

**Supplementary Table 5**

| **No.** | **Gene Symbol** | **No.** | **Gene Symbol** | **No.** | **Gene Symbol** |
| --- | --- | --- | --- | --- | --- |
| 1 | MMP2 | 63 | PTGS2 | 125 | TSPO |
| 2 | XDH | 64 | CTNNB1 | 126 | NR4A1 |
| 3 | TKT | 65 | ADH1C | 127 | IGFBP1 |
| 4 | HSD17B1 | 66 | TGFBR1 | 128 | HSPA5 |
| 5 | NOS2 | 67 | CYP2C9 | 129 | OPRD1 |
| 6 | CACNA2D1 | 68 | NOX4 | 130 | MAPK8 |
| 7 | SLC6A4 | 69 | MCHR1 | 131 | IGF1R |
| 8 | FADS1 | 70 | CCND1 | 132 | HDAC8 |
| 9 | FASN | 71 | IGFBP2 | 133 | ICAM1 |
| 10 | AKR1B10 | 72 | PRKDC | 134 | HSP90AB1 |
| 11 | CYP2D6 | 73 | ESR1 | 135 | HNF4A |
| 12 | RXRA | 74 | PFKFB3 | 136 | DRD4 |
| 13 | CYP2C19 | 75 | AGTR1 | 137 | ADH4 |
| 14 | NR1H3 | 76 | VEGFA | 138 | TERT |
| 15 | ELANE | 77 | ENPP2 | 139 | RELA |
| 16 | PTPN11 | 78 | TGFB1 | 140 | HIF1A |
| 17 | CES2 | 79 | BIRC2 | 141 | CFTR |
| 18 | HTR7 | 80 | KEAP1 | 142 | CETP |
| 19 | PLA2G2A | 81 | MGMT | 143 | NR1H4 |
| 20 | MAOA | 82 | VCP | 144 | OPRM1 |
| 21 | ALK | 83 | ACHE | 145 | CYP17A1 |
| 22 | ALDH2 | 84 | GSK3B | 146 | TLR4 |
| 23 | PON1 | 85 | MMP1 | 147 | SERPINE1 |
| 24 | SHBG | 86 | IL6 | 148 | TP53 |
| 25 | PREP | 87 | HSP90AA1 | 149 | INSR |
| 26 | BCL2 | 88 | CASP3 | 150 | TLR9 |
| 27 | CXCR2 | 89 | PPP1CA | 151 | ADH1B |
| 28 | LIPE | 90 | PARP1 | 152 | PKM |
| 29 | CYP1A1 | 91 | VDR | 153 | GCG |
| 30 | PPARG | 92 | ACE | 154 | FABP4 |
| 31 | PRKCD | 93 | PPARD | 155 | AKT1 |
| 32 | CEL | 94 | HMGCR | 156 | PIK3R1 |
| 33 | MAPT | 95 | PRKCE | 157 | ADRB3 |
| 34 | GSTP1 | 96 | ROCK1 | 158 | PABPC1 |
| 35 | CXCL8 | 97 | GSR | 159 | IDH1 |
| 36 | MAPK9 | 98 | CPA1 | 160 | PRKAB1 |
| 37 | SRD5A2 | 99 | RXRG | 161 | MIF |
| 38 | CNR1 | 100 | PRKAA2 | 162 | NFKB1 |
| 39 | NFE2L2 | 101 | HMOX1 | 163 | SIRT1 |
| 40 | NR1I3 | 102 | FLT1 | 164 | IL1B |
| 41 | MTOR | 103 | HTR1A | 165 | REN |
| 42 | CYP2A6 | 104 | ALOX12 | 166 | HSD11B1 |
| 43 | AOC3 | 105 | PTPN1 | 167 | IGFBP3 |
| 44 | GC | 106 | PRKCZ | 168 | ALB |
| 45 | PDGFRB | 107 | PPARA | 169 | CPT1A |
| 46 | CTSS | 108 | CNR2 | 170 | F3 |
| 47 | CTSB | 109 | TTR | 171 | HTR2A |
| 48 | TNF | 110 | MAOB | 172 | NR1I2 |
| 49 | MAP3K8 | 111 | CYP1A2 | 173 | AKR1A1 |
| 50 | PLA2G4A | 112 | RBP4 | 174 | P2RX7 |
| 51 | DRD2 | 113 | CTRC | 175 | CA1 |
| 52 | MPO | 114 | FTO | 176 | ERN1 |
| 53 | MPI | 115 | FABP1 | 177 | JUN |
| 54 | SCD | 116 | MDM2 | 178 | FABP2 |
| 55 | NR1H2 | 117 | NPC1L1 | 179 | CYP3A4 |
| 56 | PDPK1 | 118 | EGFR | 180 | APP |
| 57 | DGAT1 | 119 | IKBKB | 181 | COMT |
| 58 | TNFRSF1A | 120 | FFAR4 | 182 | PRKAG1 |
| 59 | STAT3 | 121 | EIF4A1 | 183 | RPS6KA3 |
| 60 | F2 | 122 | GSTK1 | 184 | SLC6A3 |
| 61 | HCAR2 | 123 | PIK3CA | 185 | PRSS1 |
| 62 | FDFT1 | 124 | GPBAR1 | 186 | MMP9 |

**Table S5.** Core gene hub of DXR in the treatment of MASH
